# Cross-genetic determination of maternal and neonatal immune mediators during pregnancy

**DOI:** 10.1186/s13073-018-0576-8

**Published:** 2018-08-22

**Authors:** Michela Traglia, Lisa A. Croen, Karen L. Jones, Luke S. Heuer, Robert Yolken, Martin Kharrazi, Gerald N. DeLorenze, Paul Ashwood, Judy Van de Water, Lauren A. Weiss

**Affiliations:** 1 Department of Psychiatry and Institute for Human Genetics, University of California, San Francisco, San Francisco, CA USA; 2 Divison of Research, Kaiser Permanente Northern California, Oakland, CA USA; 3 Department of Internal Medicine, Division of Rheumatology, Allergy, and Clinical Immunology, University of California Davis, Davis, CA USA; 4 MIND Institute, University of California Davis, Davis, CA USA; 5 Stanley Division of Developmental Neurovirology, Department of Pediatrics, Johns Hopkins University School of Medicine, Baltimore, MD USA; 6 Division of Environmental and Occupational Disease Control, California Department of Public Health, Richmond, CA USA; 7 Department of Medical Microbiology and Immunology, University of California Davis, Davis, CA USA

**Keywords:** Cytokines, Chemokines, Immune system, Maternal and fetal genetics, GWAS, SNP-based heritability, Early brain development, Autism

## Abstract

**Background:**

The immune system plays a fundamental role in development during pregnancy and early life. Alterations in circulating maternal and neonatal immune mediators have been associated with pregnancy complications as well as susceptibility to autoimmune and neurodevelopmental conditions in later life. Evidence suggests that the immune system in adults not only responds to environmental stimulation but is also under strong genetic control.

**Methods:**

This is the first genetic study of > 700 mother-infant pairs to analyse the circulating levels of 22 maternal mid-gestational serum-derived and 42 neonatal bloodspot-derived immune mediators (cytokines/chemokines) in the context of maternal and fetal genotype. We first estimated the maternal and fetal genome-wide SNP-based heritability (*h*^*2*^_*g*_) for each immune molecule and then performed genome-wide association studies (GWAS) to identify specific loci contributing to individual immune mediators. Finally, we assessed the relationship between genetic immune determinants and ASD outcome.

**Results:**

We show maternal and neonatal cytokines/chemokines displaying genetic regulation using independent methodologies. We demonstrate that novel fetal loci for immune function independently affect the physiological levels of maternal immune mediators and vice versa. The cross-associated loci are in distinct genomic regions compared with individual-specific immune mediator loci. Finally, we observed an interaction between increased IL-8 levels at birth, autism spectrum disorder (ASD) status, and a specific maternal genotype.

**Conclusions:**

Our results suggest that maternal and fetal genetic variation influences the immune system during pregnancy and at birth via distinct mechanisms and that a better understanding of immune factor determinants in early development may shed light on risk factors for developmental disorders.

**Electronic supplementary material:**

The online version of this article (10.1186/s13073-018-0576-8) contains supplementary material, which is available to authorized users.

## Background

Women experience dramatic changes in immune system status during pregnancy. Tolerance for fetal-placental antigens allowing for healthy development of the fetus must be balanced with the ability of the mother to fight infections. Further, immune dysregulation during pregnancy can lead to outcomes such as preeclampsia, fetal growth retardation, and miscarriage [1, 2]. However, little is known about inter-individual differences in immune status during pregnancy. Similarly, immune protection in early infancy is thought to be accomplished by a combination of maternal transfer and fetal production of soluble immune molecules, but the extent of maternal contribution has not been worked out for many immune molecules.

Among the soluble molecules that mediate the immune response, cytokines and chemokines are particularly important for regulating inflammation, immune cell proliferation and differentiation, and for influencing the progression of some chronic inflammatory conditions [3, 4]. Cytokines are small peptides involved in most phases of immune response, and chemokines are specific cytokines that are also important in controlling white blood cell trafficking and attracting cells to an infection site.

Immune responses to environmental stimuli (e.g., infections) are tightly regulated by genetics. Several studies have shown genetic variation associated with RNA or protein levels of immune mediators [5–7] and substantial heritability of immune cell counts [6]. Moreover, a recent study [8] identified 27 specific loci associated with circulating levels of 41 cytokines/chemokines, mainly in genes that encode the proteins and/or their receptors, and that were also associated with inflammatory and autoimmune diseases. However, despite this strong evidence for genetic control of immune system status, no study has analysed the genetic regulation of maternal immune mediator levels during pregnancy, neonatal levels at birth, or the intersection of genetic determinants of either with chronic disorders.

To fill the gaps in our current understanding of genetic regulation of the immune system during pregnancy and at birth, we first hypothesize that maternal mid-gestational mediator levels will be regulated by maternal genetics, some of which could be unique to pregnancy. Second, we define two possible scenarios for neonatal immune system status. At birth, the neonatal immune system might be at least partially determined by the mother; so, we hypothesize that maternal genetics could regulate not only maternal mediator levels but also contribute to neonatal mediator levels. In the second scenario, we hypothesize that the neonatal genome would exert independent influence on neonatal mediator levels.

Increasing evidence shows that during pregnancy, fetal genetics also contributes to different aspects of maternal physiology, such as blood pressure, gestational diabetes, metabolism, and preeclampsia [9–11]. We previously showed that some maternal circulating toxicant levels in pregnancy are in part regulated by fetal genetics [12]. However, there has been no study looking at the potential fetal genetic influences on maternal immune status during pregnancy. Thus, we also hypothesize that genetic variation in the fetus and placenta might influence the mother’s immune system function during pregnancy.

Understanding patterns of genetic regulation of circulating maternal and neonatal immune mediators might elucidate important mechanisms for immune system status and disease susceptibilities. Beyond their classical roles in immune function, recent evidence suggests that some cytokines show pleiotropic effects in the central nervous system (CNS), acting as neuromodulators, growth and survival factors [13], neurodevelopmental organizers [14], and ultimately influencing behavior and cognition. Animal models [15] of maternal immune activation (MIA) during pregnancy demonstrate behavioral abnormalities in offspring, proposed to be mediated via cytokines [16–18]. In addition, peripheral abnormalities of circulating cytokine levels have been observed in individuals affected by neuropsychiatric disorders, such as major depression, bipolar disorder, schizophrenia, and autism spectrum disorder (ASD) [19–23].

Among neurodevelopmental disorders, ASD is thought to originate in early development. ASD is a highly heritable complex disease, with both genetic and non-genetic risk factors, proposed to include maternal infection [24] and fever [25, 26] and maternal and neonatal immune system dysregulation [27–30]. Elevated peripheral cytokine profiles in pregnancy [31] and at birth have been associated with ASD diagnosis in childhood [32, 33]. A study in our Early Markers for Autism (EMA) maternal dataset found elevated maternal cytokine levels during mid-gestation associated with an increased risk of ASD with intellectual disability [34]. We have also observed elevated levels of cytokines/chemokines in neonatal bloodspots from ASD-affected children compared to controls. However, neither our studies nor others implicating immune mediators in ASD have included measurement of genetics to distinguish inborn from environmentally stimulated variation in immune molecules.

To test our hypotheses, we applied several methodologies to a large set of maternal and neonatal soluble immune mediators (SIMs), specifically cytokines and chemokines, in combination with genetic markers in the EMA cohort, a population-based nested case-control study of ASD. This dataset utilizes samples from a maternal prenatal screening program and neonatal bloodspots from the sampled pregnancies to measure genetic and immune molecules (with no direct measurement or record of infection or illness). (Note that we define the genetic contribution from the fetus during pregnancy and from the neonate at birth as ‘fetal genetics’ for consistency of terminology and to represent the likely timing of genetic regulation, although the genetic data were collected shortly after birth). We first estimated heritability to determine the extent to which an individual’s cytokine/chemokine levels might be genetically regulated. We next performed genome-wide association identifying specific contributing loci. We also used several approaches to investigate whether neonatal cytokines/chemokines might be influenced by maternal genetic variation and/or maternal cytokines/chemokines could be influenced by fetal genetic variation. Finally, in order to understand the intersection of genetic determinants of maternal and/or fetal immune function with developmental disorders, we studied the potential for interaction between an immune mediator and genetic variation on ASD outcome.

## Methods

### Study population and blood sampling

The Early Markers for Autism (EMA) study is a population-based nested case-control study [35, 36] that includes a population of pregnant women (15–20 weeks) and their babies from Orange, San Diego and Imperial Counties, California, who were enrolled in the State’s Prenatal Expanded Alphafetoprotein Screening Program and delivered a live-born infant in 2000–2003. We used prenatal (maternal blood) and newborn (neonatal blood spot) specimens from each mother-baby matching pair. The offspring outcome of ASD was ascertained from the client files of two regional centers (RCs) and verified by study clinician expert review of records according to a protocol developed by the Metropolitan Atlanta Developmental Disabilities Surveillance Program, as described previously [12, 36]. The controls were randomly sampled from the birth certificate files after past or current RC clients had been excluded, matched to ASD cases by sex, birth month, and birth year [36]. Maternal blood samples were collected in serum separator tubes at 15–20 weeks gestation and stored as part of Project Baby’s Breath. Serum was stored in cryovials and cell pellets stored in SSTs at − 20 °C. Newborn blood spots were collected on filter paper 1–2 days after birth and stored at − 20 °C and maintained by the Genetic Disease Screening Program, California Department of Public Health.

### Cytokine and chemokine measurement

Maternal mid-gestational serum concentrations of 22 cytokines and chemokines were determined using a commercially available multiplex bead-based kit (MILLIPLEX MAP Human Cytokine/Chemokine Kit; Millipore, Billerica, MA, USA) in accordance with the kit-specific protocols provided by Millipore and as already described [34] (see more details in Additional file 1: Supplemental methods). We measured 16 cytokines and six chemokines reported in Additional file 2: Table S1. The neonatal levels of peripheral blood immune markers were extracted from filter spots as described in Additional file 1: Supplemental methods and determined using a commercially available, slightly modified, Luminex multiplex assay. We combined a Bio-Plex Pro Human Chemokine kit (Bio-Rad, Hercules, CA) containing a mix of 40 different immune markers with two individual single-plex beads from the same company, interleukin (IL)-12p70 and IL-13 (see more details in Additional file 1: Supplemental methods). We measured 12 cytokines and 30 chemokines reported in Additional file 2: Table S1. All study procedures were approved by the institutional review boards of the California Health and Human Services Agency and Kaiser Permanente Northern California; it was determined at UCSF Committee on Human Research that the institution was not engaged in human subject research.

### DNA extraction and genotyping

The QIAGEN QIAamp 96 DNA Blood Kit was used to extract DNA from a subset of maternal and neonatal blood samples and the Invitrogen Quant-iT DNA Assay Kit to measure the DNA concentration by the biomedical laboratory at Utah State University, as previously described [36]. Maternal and neonatal samples were genotyped using the Affymetrix Axiom (Affymetrix 2011) EUR array (675,000 SNPs across the genome) by the Genomics Core Facility (GCF) at UCSF, using standard protocols. Genotype calling was carried out using Affymetrix Power-Tools (‘Affymetrix Power Tools, Affymetrix Website’), as previously described [36]. Individual-based and marker-based quality controls, such as detection of Mendelian errors and HWE assessment, were performed with PLINK software [37] as reported in Tsang et al. [36]. Additionally, we extracted only common SNPs (MAF ≥ 1%). Two high quality datasets were used in our analysis: the first dataset included 790 maternal samples of varied ancestry (390 ASD cases, 400 controls) and 629,686 genotyped markers, and the second dataset included 764 neonatal samples (385 ASD cases, 379 controls) and 622,716 genotyped markers. Most of these were related pairs of maternal-neonatal samples (366 case pairs, 369 control pairs). The maternal dataset was a subset of those with immune mediator levels reported in Jones et al. [34].

### Ancestry analysis

We reported in Traglia et al. [12] the genome-wide multidimensional scaling (MDS) analysis on high-quality markers genotyped in mothers and in infants included in our dataset. The resulting maternal and fetal genetic matrices included ten principal coordinates that summarized the genetic distance between each maternal and neonatal sample and that captured 90% and 89% of the maternal and fetal genetic variance. The distribution of maternal race/ethnicity based on the birth certificate was: 42% Hispanic, 35% non-Hispanic Caucasian, 15% Asian, 3% South Asian, and 3% African American. The ten maternal and fetal principal coordinates showed highly significant pairwise correlation with *ρ* ranging between 0.82 and 0.95 (Spearman’s test in R 3.2.0 environment [38] *P* < 0.05; Additional file 2: Table S2).

### Confounding factors for maternal and neonatal immune mediator levels and linear correlations

**Fig. 1:**
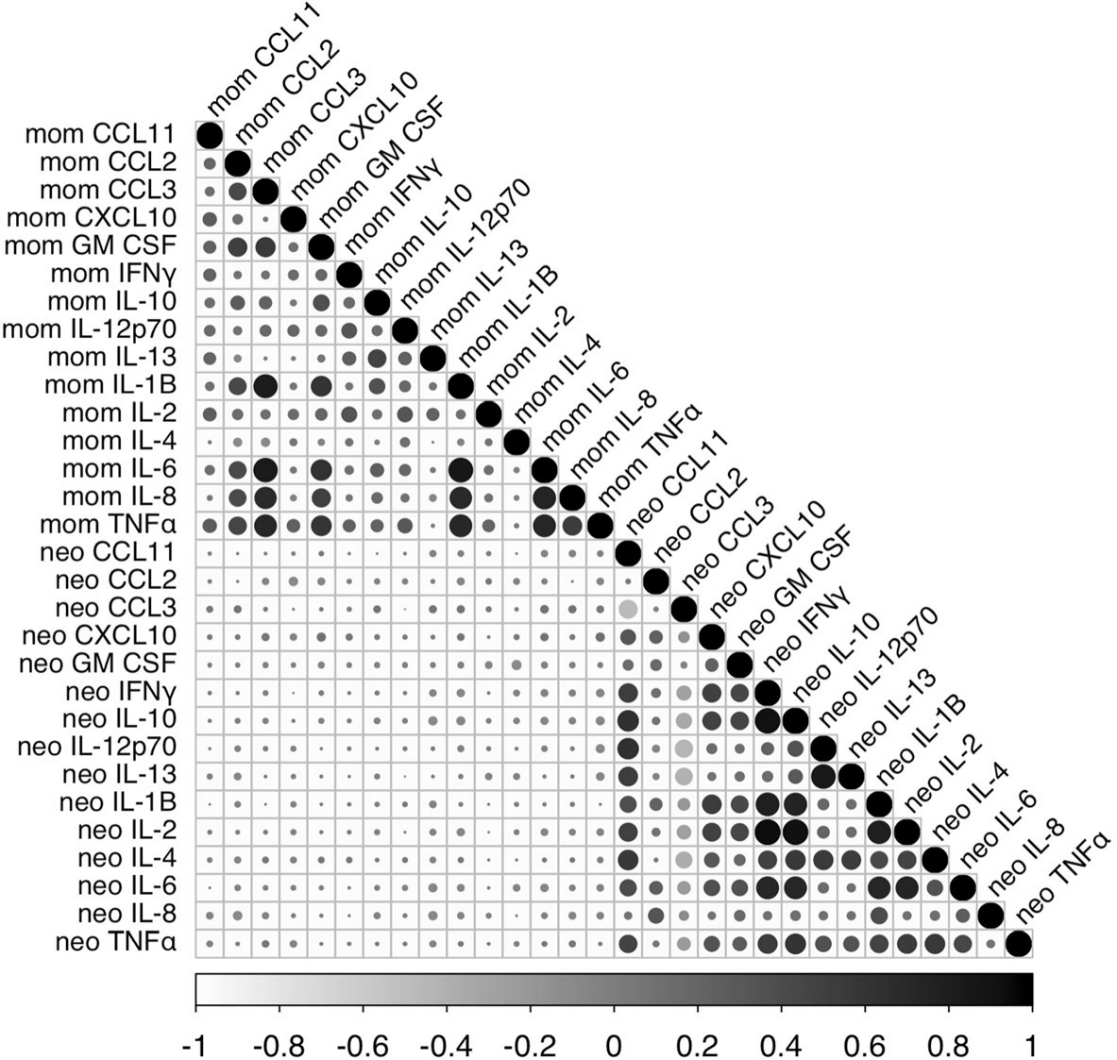
Linear non-parametric correlation coefficients for 15 overlapping maternal and neonatal cytokines and chemokines. Correlation coefficients were calculated with Spearman’s test. From weakest to strongest, positive correlation coefficients are represented in a dark grey gradient; negative correlation coefficients are displayed in a light grey gradient

We analysed the log-transformed levels of the 22 maternal and 42 neonatal cytokines and chemokines with > 60% of values greater than their limit of detection (LOD) defined as the fluorescence intensity signal 2 standard deviations above the background signal, as reported in the assay manual (see Additional file 1: Supplemental methods and Additional file 2: Table S1). The values below LOD were replaced with *LOD/√2* before the normalization, as reported in Jones et al. [34]. We applied a threshold of 3 or 4 SD from the mean to exclude outliers. The number of extreme values is reported in Additional file 2: Table S3. We analysed the effects of available socio-demographic and technical covariates on maternal and neonatal immune mediator levels with linear regression models in the genotyped mothers and infants using R 3.2.0 [38]. In all the maternal analyses, we included potential confounding factors that were nominally associated with at least three maternal immune mediators (*P* < 0.05), such as maternal country of birth (USA, Mexico, others), age at mid-pregnancy (15–45 years old), maternal gestational days and weight, year of birth, maternal educational attainment (elementary, high school, college, post-graduate), and maternal genetic ancestry (the first ten coordinates) (Additional file 3: Figure S1). No variables nominally significant in one immune mediator (0.01 < *P* < 0.05) would survive multiple testing correction. We also used confounding factors associated with at least three neonatal immune mediators in all the neonatal analyses: offspring sex, bloodspot time after birth, birth type (spontaneous, C-section) and weight, birth month and year, maternal gestational days, maternal and paternal age and educational attainment, number of prenatal visits, number of previous live births, assay plate number, neonatal TSH levels, and neonatal genetic ancestry (1–10 coordinates) (Additional file 3: Figure S1). In both datasets, we used offspring ASD status as an additional covariate for quantitative cytokine analyses. The statistics for the residual levels of each cytokine/chemokine after adjustment for confounding factors are reported in Additional file 2: Table S3. Finally, we performed a Shapiro-Wilk normality test in the R 3.2.0 [38] and reported in Additional file 2: Table S3. We assessed the linear correlation across 22 maternal cytokines/chemokines and 42 neonatal cytokines/chemokines, including 15 overlapping molecules measured in both datasets, using the Spearman’s test implemented in R 3.2.0 ‘corrplot’ package (Fig. 1, Additional file 2: Tables S4, S5, and S6).

### SNP-based heritability and genetic correlation

The final set of maternal and fetal autosomal high quality markers were used to generate genetic relationship matrices and calculate SNP-based additive heritability (*h*^*2*^_*g*_) for each maternal and neonatal cytokine/chemokine level with a Restricted Maximum Likelihood (REML) model implemented in GCTA software [39], taking into account each specific set of covariates. The heritability estimation indicates the proportion of the total phenotypic variance of each cytokine/chemokine level (*σ*_*p*_) accounted for by the genetic variance (*σ*_*g*_*/σ*_*p*_) after the exclusion of the effect of the sociodemographic, ancestry, and case/control status covariates. For each maternal and neonatal cytokine/chemokine, we used both maternal and fetal genotypes to assess the heritability for the same cytokine/chemokine. We estimated that our dataset has low power to detect moderate heritability via the REML approach with GCTA-GREML Power Calculator [40]. To validate that our approach is suitable to adjust for population stratification, we used permutations swapping the phenotype labels for each individual 100 times, and we estimated 100 SNP-based permuted heritability models for the significantly heritable immune mediators. Most of the significant maternal and fetal *h*^*2*^_*g*_ are in the top 5% of the permuted *h*^*2*^_*g*_ distributions, and the medians are close to zero, suggesting little bias from population structure. To determine whether the significantly heritable maternal and neonatal cytokines/chemokines shared genetic determinants, we estimated the genetic correlations (*r*_*g*_) between all possible pairs within the two datasets using the estimated heritability (*h*^*2*^_*g*_) for each heritable immune mediator in a bivariate REML [41] model in GCTA software [39] and compared to the linear correlations across each pair with cor.test() in the R 3.2.0 ‘stats’ package [38]. We applied Fisher’s z transformation test in the R 3.2.0 environment [38] to assess significant differences between estimated correlation coefficients (Additional file 2: Table S7). For each cytokine/chemokine that shows both significant maternal and fetal heritability, we assessed whether both individuals independently contribute to the phenotypic variance. We used PLINK software [37] and ad hoc bash scripts to compare each maternal allele to the matching fetal allele for the same locus (*a1a2*), and we replaced each maternal genotype with the one maternal allele not transmitted to offspring and each fetal genotype with one allele not inherited from the maternal lineage (i.e. for SNP1 maternal *a1a2* = AA and fetal *a1a2* = AG, A is the maternal non-transmitted allele and G is the fetal non-maternal allele). We set to 0 (missing genotype) all the SNPs for which we were unable to determine which allele was maternal (heterozygous genotype in both mother and offspring). This approach may affect the estimate of the genetic contribution by introducing missing genotypes among the more common SNPs; thus, we interpret having significant heritability as meaningful but do not interpret the estimates as precise. We used PLINK software [37] to merge the individual pairs and create two new sets of independent maternal and fetal genotypes (--make-bed). In the genetic analysis, we wanted to take into account the haploid status, so we also replaced the sex of all individuals with 1 (male) and the chromosome with X (--update-sex; --update-chr), and we coded the entire maternal and fetal independent haploid genomes as male pseudo-X chromosomes. Two genetic matrices that include only the maternal-specific alleles and the fetal-specific alleles and that look like X-chromosomes were generated from PLINK bed files with GCTA software [39] using a no dosage compensation (ND) model (--dc 0; --make-grm-xchr) and used in cytokine-specific REML models. The ND model assumes that each allele has a similar effect on the trait. The genetic relationship matrix (A_X_) for the X chromosome is redefined as A_X_^ND^ = 1/2A_X_ for male-male pairs [39]. We applied the Fisher scoring approach as implemented in GCTA (--reml alg 1) for models that did not converge. It should be noted that the estimates might be overestimated by using only one haploid genetic matrix in the model in addition to the low power of moderate heritability detection. Thus, in the text, these estimates are not considered as ‘heritability’ in a classic sense, but, when significant, as an indication of maternal and/or fetal genetic contribution to the trait.

### Genome-wide association study

We performed immune mediator-specific genome-wide association studies (GWAS) for maternal and fetal SNPs using a linear model implemented in PLINK software [37] (--linear) using each specific set of covariates including ten PCs. To validate that our approach is suitable to adjust for stratification ‘within’ and ‘across’ populations, we also performed a meta-analysis of four separate association tests in homogeneous sub-populations with ten population-specific PCs and we found consistent results for top SNPs (Additional file 2: Table S8). We also extracted the meta-analysis top hits (*P* < 5 × 10^−8^) and we observed a similar trend (Additional file 2: Table S8). Then, we used the LocusZoom tool [42] to generate regional genomic plots and assess linkage disequilibrium (LD) among associated SNPs. We use the genome-wide significance threshold (*P* < 5 × 10^−8^) and suggestive threshold (*P* < 1 × 10^−7^) to account for approximate independent common polymorphism testing per GWAS [43, 44]. We have tested many correlated cytokines/chemokines in related mothers and offspring; because the tests are not completely independent, we were unable to calculate the exact correction for study-wide significance and present uncorrected *P* values.

### Maternal and fetal contribution to the associated loci

For each cytokine/chemokine that showed a genome-wide significant maternal and/or fetal locus that is also suggestively associated in the paired individual’s genetics, we assessed whether the associations were controlled by maternal and/or fetal genetics. We performed separate linear regression models including the entire maternal- and/or fetal-specific set of covariates and the genotypes of maternal- and fetal-associated SNPs (for three maternal immune mediators ~ maternal SNP + fetal SNP + maternal covariates; for ten neonatal immune mediators ~ fetal SNP + maternal SNP + fetal covariates), and we assessed the residual association for maternal and fetal genotypes.

### Offspring ASD outcome association with immune mediators

We tested for differences between the residuals for maternal cytokines/chemokines in mothers of ASD cases and mothers of controls and for neonatal cytokines/chemokines in ASD-affected neonates and control neonates with a two-sample Mann-Whitney Wilcoxon test in R 3.2.0 [38].

### Genetic interaction between chemokine levels and ASD association

We assessed whether genetic determinants associated with chemokines that showed statistically significant associations with ASD might drive the association between chemokines and ASD or show interaction effects. Thus, we selected each maternal and fetal cytokine-specific top (GW or suggestive) SNPs for maternal and neonatal chemokines that are associated with ASD. We included each genotype in logistic regression models for ASD outcome and we assessed whether the top SNPs interact with ASD in cytokine-specific models. We compared the levels of chemokines in the individuals with different genotypes for ASD-interacting SNPs using a two-sample Mann-Whitney Wilcoxon test in the R 3.2.0 environment [38].

## Results

### Maternal immune mediators

To assess our ability to identify genetic contributions of circulating maternal immune mediators, we first analysed distributions of the log-transformed concentrations of 16 cytokines and six chemokines surveyed in mid-gestational maternal blood. We applied adjustment for potential maternal and neonatal confounding factors, including offspring ASD outcome (Additional file 3: Figure S1) to obtain residuals, as we first wanted to assess cytokine/chemokine levels independent of outcome. After excluding a few extreme residual values for 11 of 22 mediators (outliers < 4% of the total individuals, mean = 1%; see the ‘Methods’ section), we observed approximately normally distributed residuals for most cytokine/chemokine levels (Additional file 2: Table S2). The observation of unimodal distributions suggested continuous inter-individual variation in immune mediator levels, rather than distinct classes of individuals with low/high levels, such as immune-activated vs. non-activated, within the study population. Thus, all 22 maternal cytokines and chemokines could be used as quantitative traits for genetic analysis. Some maternal immune mediators appeared interrelated, as 22 of 231 pairs (9.5%) demonstrated high correlations (*ρ* > 0.5) (Additional file 2: Table S4).

### High maternal heritability contributes to two maternal immune mediator levels

We estimated SNP-based maternal heritability for 22 maternal mid-gestational mediator residual levels via mixed linear model. CXCL10 (or IP-10) and IL-7 were significantly regulated by genome-wide maternal SNP effects (Table 1). The additive maternal polygenic contribution is estimated to account for 79% and 84% of the total CXCL10 and IL-7 phenotypic variance (*P* = 4.4 × 10^−3^ and *P* = 2.1 × 10^−3^, respectively). We are not able to determine whether the remaining immune mediators might be heritable because our dataset has low power to detect moderate heritability.

**Table 1:** SNP-based maternal and fetal heritability for maternal and neonatal chemokines and cytokines

SIM	Dataset	Maternal genetics			Fetal genetics		
		*h* ^*2*^_*g*_	SE	*P* value	*h* ^*2*^_*g*_	SE	*P* value
Chemokines							
CXCL10	Mothers	0.79	0.28	4.4 × 10^−3^	0.99	0.38	5.8 × 10^−4^
CCL1	Infants	0.72	0.35	2.9 × 10^−2^	0.98	0.35	1.6 × 10^− 2^
CCL3	Infants	0.60	0.33	4.6 × 10^−2^	0.72	0.43	5.0 × 10^−2^
CCL17	Infants	0.59	0.37	0.09	0.99	0.42	1.0 × 10^−2^
CCL19	Infants	0.69	0.36	4.0 × 10^−2^	0.99	0.42	7.8 × 10^−3^
CCL22	Infants	0.92	0.32	3.8 × 10^−3^	0.97	0.43	2.0 × 10^−2^
CCL25	Infants	0.50	0.33	0.07	0.70	0.42	5.0 × 10^−2^
CXCL5	Infants	0.88	0.34	1.4 × 10^−2^	0.92	0.43	2.0 × 10^−2^
Cytokines							
IL-4	Infants	0.76	0.37	3.2 × 10^−2^	0.05	0.49	NS
IL-7	Mothers	0.84	0.28	2.1 × 10^−3^	0.90	0.37	1.0 × 10^−2^

*NS* not significant

### Maternal loci associated with maternal mediators

We performed genome-wide association studies (GWAS) for maternal cytokine/chemokine levels via linear regression. The maternal cytokines sIL-2Ra and IL-1α and the chemokine CCL11, were significantly (*P* < 5 × 10^−8^) associated with specific maternal loci (Table 2 and Additional file 3: Figure S2A–C). CCL11 (or eotaxin-1) is associated with a low-frequency polymorphism, rs115463265, which maps on chromosome 3p24.2 in a lincRNA between *THRB* and *RARB* genes encoding receptors for thyroid hormone. Soluble IL-2Ra is associated with rs12778662, an intronic variant located in a RNA binding protein gene (*RBM17*) and near the gene encoding the IL-2 receptor (*IL2RA*) on chromosome 10p15.1. Finally, IL-1α is associated with rs1562064 near the *SMAD1* gene which encodes a member of the bone morphogenetic protein (BMP) pathway on chromosome 4q31.21.

**Table 2:** Maternal and fetal genome-wide significant association of maternal and neonatal cytokines/chemokines

SNP	gen	chr	A1	MAF	Beta	SE	*P* value	Locus	SIM	Set	*P* value match-gen
rs12327057	Fetal	18p11	C	0.18	− 0.42	0.07	1.4 × 10^−8^	*ADCYAP1*	sIL2R-α	M	9.4 × 10^−4^
rs75885714	Fetal	3p24.3	C	0.08	− 0.53	0.05	8.6 × 10^−21^	*PLCL2*	CCL17	I	3.8 × 10^−6^
rs75885714	Fetal	3p24.3	C	0.08	− 0.48	0.05	3.3 × 10^−20^	*PLCL2*	CCL19	I	3.8 × 10^−6^
rs75885714	Fetal	3p24.3	C	0.08	− 0.36	0.04	2.8 × 10^−19^	*PLCL2*	CXCL9	I	5.4 × 10^−6^
rs75885714	Fetal	3p24.3	C	0.08	− 0.23	0.03	8.2 × 10^−13^	*PLCL2*	CCL7	I	1.1 × 10^−3^
rs75885714	Fetal	3p24.3	C	0.08	− 0.24	0.03	5.2 × 10^−12^	*PLCL2*	IFN-γ	I	6.0 × 10^−3^
rs75885714	Fetal	3p24.3	C	0.08	− 0.27	0.04	2.1 × 10^−12^	*PLCL2*	IL-2	I	1.5 × 10^−3^
rs75885714	Fetal	3p24.3	C	0.08	− 0.26	0.04	1.7 × 10^−11^	*PLCL2*	IL-6	I	9.7 × 10^−4^
rs75885714	Fetal	3p24.3	C	0.08	− 0.20	0.03	1.8 × 10^−11^	*PLCL2*	IL-10	I	3.8 × 10^−4^
rs75885714	Fetal	3p24.3	C	0.08	− 0.22	0.03	3.5 × 10^−10^	*PLCL2*	IL-1β	I	1.4 × 10^−3^
rs75885714	Fetal	3p24.3	C	0.08	− 0.17	0.03	9.3 × 10^−10^	*PLCL2*	CXCL13	I	1.3 × 10^−4^
rs75885714	Fetal	3p24.3	C	0.08	− 0.16	0.03	2.0 × 10^−8^	*PLCL2*	CX3CL1	I	9.8 × 10^−4^
rs1003645	Fetal	17q12	C	0.28	− 0.63	0.02	2.5 × 10^−100^	*CCL23*	CCL23	I	3.4 × 10^−24^
rs854625	Fetal	17q12	A	0.12	0.47	0.04	4.6 × 10^−25^	*CCL15*	CCL15	I	1.1 × 10^−6^
rs3921	Fetal	4q21.1	C	0.29	0.24	0.03	2.0 × 10^−14^	*CXCL9/10/11*	CXCL11	I	1.2 × 10^−3^
rs16850073	Fetal	4q13.3	T	0.32	0.10	0.02	6.4 × 10^−9^	*CXCL6*	CXCL6	I	6.8 × 10^−6^
rs73359750	Fetal	7q11.23	T	0.08	0.86	0.16	4.9 × 10^−8^	*CCL24*	CCL24	I	NS
rs41272321	Fetal	3q22.1	G	0.13	− 0.20	0.03	2.8 × 10^−10^	*ACKR4*	CCL21	I	3.3 × 10^−4^
rs2228467	Fetal	3p22.1	C	0.04	0.29	0.05	2.8 × 10^−8^	*ACKR2*	CXCL9	I	0.01
rs2228467	Fetal	3p22.1	C	0.04	0.42	0.07	4.4 × 10^−9^	*ACKR2*	CCL19	I	0.03
rs2228467	Fetal	3p22.1	C	0.04	0.48	0.07	1.8 × 10^−10^	*ACKR2*	CCL17	I	0.02
rs74331971	Fetal	8p23.3	A	0.03	− 0.40	0.07	1.9 × 10^−9^	*FBXO25/TDRP*	IL-4	I	0.03
rs4303899	Fetal	3q13.32	G	0.12	− 0.09	0.02	1.7 × 10^−8^	lincRNA	CXCL12	I	NS
rs115463265	Maternal	3p24.2	T	0.02	− 1.50	0.24	1.6 × 10^−9^	*THRB/RARB*	CCL11	M	NS
rs12778662	Maternal	10p15.1	T	0.07	0.65	0.11	3.7 × 10^−9^	*IL2R*	sIL2R-α	M	0.04
rs1562064	Maternal	4q31.21	G	0.32	− 0.74	0.13	2.1 × 10^−8^	near *SMAD1*	IL1-α	M	1.5 × 10^−3^
rs34642455	Maternal	7q22.1	C	0.13	− 2.17	0.38	3.1 × 10^−8^	*CYP3A4*	CXCL5	I	3.6 × 10^−3^
rs72751339	Maternal	15q26.2	T	0.02	− 1.52	0.25	1.3 × 10^−8^	*MCTP2*	CCL24	I	1.7 × 10^−3^
rs17159338	Maternal	5q21.3	C	0.03	− 0.36	0.06	1.2 × 10^−9^	*near EFNA5*	IL-16	I	NS

*gen* genetics, *chr* chromosomal region, *A1* tested allele, *MAF* minor allele frequency, *Set* dataset, *M* mothers, *I* infants and *NS* not significant

### Neonatal immune mediators

We analysed the log-transformed concentrations of 12 cytokines and 30 chemokines measured in neonatal bloodspots at birth in the genotyped EMA sample. As reported in Additional file 2: Table S2, 15 out of 42 cytokines/chemokines measured in the neonates overlapped with those measured in maternal serum. After applying adjustment for a set of maternal and neonatal confounding factors (described in the ‘Methods’ section) and exclusion of extreme values (outliers < 7% of the total individuals, mean = 1%, Additional file 2: Table S3 and Additional file 3: Figure S1), neonates showed significantly less variance than mothers for the immune mediators measured in both individuals (F test, *P* < 0.05) and particularly tight but approximately normal distributions and high correlations among most of the immune mediators (Additional file 2: Tables S3 and S5). We did not observe any significant correlation (or coefficients *ρ* > 0.15) between the 15 cytokines/chemokines measured in the maternal dataset and the same 15 mediators measured in the neonatal dataset (Fig. 1). Nor did we find other correlated maternal-neonatal immune mediator pairs, considering all combinations in case we could infer a relationship with a non-measured cytokine/chemokine (Additional file 2: Table S6).

### High fetal heritability regulates the levels of seven neonatal chemokines

In order to assess whether the infant immune mediator levels measured at birth might be genetically regulated independently of the maternal immune system, we measured SNP-based fetal heritability for 42 neonatal cytokine/chemokine levels after adjustment for potential confounding factors (including ASD status) via mixed linear modelling. We were able to identify seven neonatal chemokines that showed significant fetal genetic contribution (heritability = 70–99%; Table 1). To assess whether the subset of heritable neonatal chemokines shared genetic determinants, we calculated the co-heritability (genetic correlation) between all the possible pairs of the seven neonatal chemokines, and we compared genetic correlations to linear correlations. We expected that genetic correlation might be higher than linear correlation if there were differences in environmental determinants but similar genetic determinants of both immune mediators. In contrast, significantly lower genetic correlations than linear correlations might indicate a similar response to the shared environment but distinct genetic determinants involved. We found seven out of 21 chemokine pairs that showed significant genetic correlations (*ρ* > 0.50) and four were significantly higher compared to the linear correlations (Additional file 2: Table S7). Thus, our results allowed us to define a set of neonatal immune mediators with strong genetic control that includes CCL1, CCL3, CCL17, CCL19, CCL22, and CCL25. Our results support the hypothesis that some neonatal chemokines are under strong fetal genetic control early in life.

### PLCL2 is a novel fetal locus for several neonatal immune mediators

**Fig. 2:**
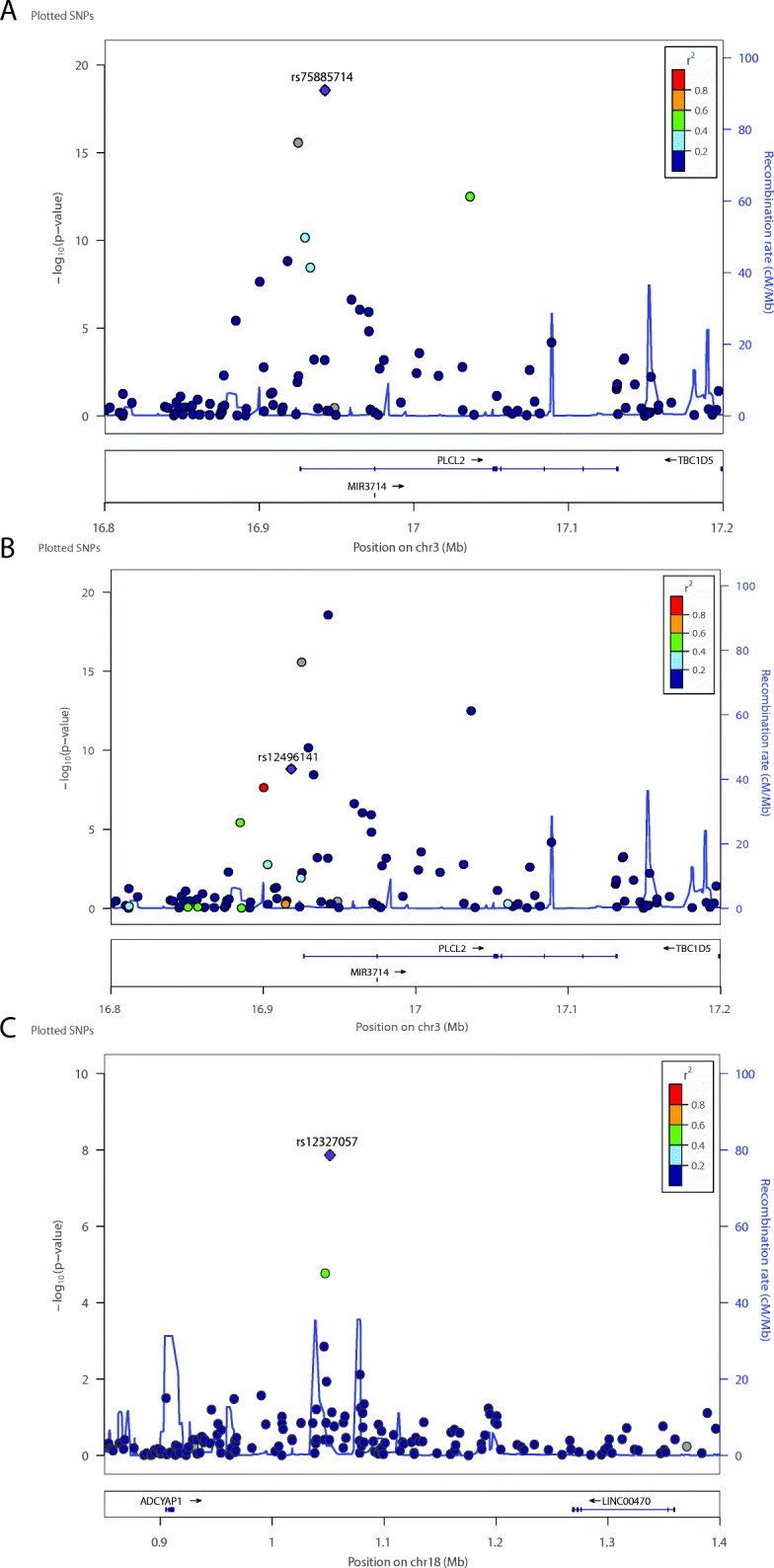
Linkage disequilibrium regional genomic plot of fetal genome-wide associated SNPs with maternal serum levels of sIL2R-α and 11 neonatal immune mediators. **a** Fetal rs75885714 on chromosome 3p24.3 associated with CCL17 (*β* = −0.53, SE = 0.05, *P* = 8.6 × 10^−21^); **b** The independent fetal SNP rs12496141 which maps in the *PLCL2* gene and (**c**) Fetal rs12327057 on chromosome 18p11 associated with sIL2R-α maternal levels (*β* = − 0.42, SE = 0.07, *P* = 1.4 × 10^−8^) maps to a lincRNA *ADCYAP1* gene. The x-axis represents the genomic position; the y-axis shows the negative logarithm of the observed association *P* value for each tested SNP. Plotted with LocusZoom tool [42]

Next, we performed genome-wide association studies for the 42 neonatal cytokine/chemokine levels via linear regression analyses. We observed a SNP (rs75885714, MAF = 7%) located on chromosome 3p24.3 that was highly associated with 11 neonatal cytokines and chemokines (Table 2 and Additional file 2: Table S5). The strongest association was with the chemokine CCL17 (or TARC, *β* = − 0.53, SE = 0.05, *P* = 8.6 × 10^−21^). This SNP was also associated with a number of inflammatory cytokines and chemokines: IFNγ**,** IL-2, CCL7, CXCL9, and CCL19. We next asked whether the rs75885714 locus is independently associated with each immune mediator or whether it drives the regulation of the entire set of correlated mediators. Regression models including CCL17 with each other cytokine/chemokine separately suggest that CXCL9 might independently account for the association between rs75885714 and the other correlated cytokines/chemokines. To confirm this, we performed a linear regression model for CXCL9 after including the entire set of ten correlated cytokines/chemokines and we still observed a residual association between rs75885714 and CXCL9. Thus, the association of the locus with the cytokines/chemokines is driven by CXCL9 (rs75885714, *β* = − 0.36, SE = 0.04, *P* = 2.8 × 10^−19^), for which the SNP was responsible for 13% of the total CXCL9 phenotypic variance. The polymorphism rs75885714 maps to the *PLCL2* gene, which encodes phospholipase C (Fig. 2a). A second SNP in the same locus (rs12496141 CXCL9, *β* = − 0.21, SE = 0.03, *P* = 1.5 × 10^−9^; MAF = 10%; variance explained = 19%) shows high linkage disequilibrium (LD) with rs75885714 in our dataset (*r*^2^ = 0.68, comparable to LD estimated in Hispanic ancestry populations included in 1000 Genomes Project [45] with ENSEMBL [46]; Fig. 2b). After a conditional analysis using the individual genotypes of rs75885714 as covariate, the second SNP, rs12496141, showed no association.

A large set of other neonatal immune mediators was associated with specific fetal loci. Most of the genome-wide significant loci were near the gene encoding the associated neonatal cytokine/chemokine or its receptor (Table 2 and Additional file 3: Figure S2D–H). Additionally, we found an association with *ACKR4* (atypical chemokine receptor 4) and *ACKR2* (atypical chemokine receptor 2) loci encoding receptors that serve several chemokines (Table 2 and Additional file 3: Figure S2I–J).

We identified two additional novel loci genome-wide significantly associated with immune molecule levels (Table 2 and Additional file 3: Figure S2K–L) not near molecule- or receptor-encoding genes: IL-4 levels were associated with the low-frequency SNP rs74331971 that maps between *FBXO25* and *TDRP* on chromosome 8p23.3 (*β* = − 0.40, SE = 0.07, *P* = 1.9 × 10^−9^), and the levels of CXCL12 (or SDF-1) were associated with rs4303899, located in LINC02024, a long non-coding RNA on chromosome 3q13.32 between *LSAMP* and *IGSF11* (*β* = − 0.09, SE = 0.02, *P* = 1.7 × 10^−8^).

### Neonatal immune mediators show evidence for maternal heritability

We performed similar genetic analyses using the 42 neonatal cytokine/chemokine levels, but instead of fetal genotype data, we substituted maternal genotype data. We defined the cytokines and chemokines measured in the neonatal dataset with maternal genetic contribution and vice versa as ‘cross-heritable’. Six neonatal cytokines and chemokines showed significant contribution of maternal heritability, ranging between 60% for CCL3 (or MIP-1α) and 92% for CCL22 (or MDC). Five of these six were also neonatally heritable; additionally, the neonatal level of the cytokine IL-4 was not influenced by fetal genetics but only by maternal heritability (Table 1). The high standard error did not allow us to distinguish whether both maternal and fetal genomes contribute independently or only one individual’s genome exerts influence (but the other shares 50% of alleles by inheritance).

To differentiate between the contributions of the maternal and neonatal genome, we identified the offspring alleles not inherited from the maternal lineage at conception (fetal-specific alleles) and the maternal alleles not transmitted to the offspring (maternal-specific alleles) (see the ‘Methods’ section) and we calculated genetic contribution using maternal-specific alleles and fetal-specific alleles. (Note that we are using haploid genomes, so significant values are interpreted as evidence for independent genetic contribution rather than as a traditional ‘heritability’ estimate). We observed that three out of six cross-heritable neonatal cytokine/chemokine levels were significantly estimated as controlled by both maternal and fetal genetics, as well as CXCL5 only by maternal genetics (CCL1 and IL-4 estimate were not significant; Table 3).

**Table 3:** Maternal-specific allele-based proportion of genetic variance and fetal-specific allele-based proportion of genetic variance for maternal and neonatal chemokines and cytokines under a no dosage compensation model

SIM	Dataset	Maternal-only non transmitted allele			Fetal-only non inherited allele		
		*V*_*(g)*_ */Vp*	SE	*P* value	*V*_*(g)*_ */Vp*	SE	*P* value
Chemokines							
CXCL10	Mothers	0.81	0.19	7.1 × 10^−3^	0.81	0.20	0.01
CCL1	Infants	0.63	0.31	*0.09*	0.75	0.26	0.04
CCL3	Infants	0.47	0.40	NS	0.41	0.44	NS
CCL17	Infants	0.75	0.25	0.05	0.81	0.22	0.02
CCL19	Infants	0.80	0.23	0.03	0.83	0.21	0.01
CCL22	Infants	0.95	0.16	2.8 × 10^−3^	0.83	0.22	0.02
CCL25	Infants	0.48	0.37	NS	0.39	0.44	NS
CXCL5	Infants	0.87	0.20	0.025	0.45	0.41	NS
Cytokines							
IL-4	Infants	0.49	0.40	NS	0.00	0.73	NS
IL-7	Mothers	0.79	0.21	0.0163	0.99^#^	0.17	< 0.01

*V*_*(g)*_*/Vp* proportion of genetic variance over phenotypic variance

^#^Fisher’s test applied for non converging models

*NS* not significant

### Maternal genome-wide associated loci for the levels of three neonatal immune mediators

In order to determine whether maternal variation in the cytokine/chemokine- or receptor-encoding genes associated with maternal cytokine/chemokines also contributed to neonatal cytokine/chemokine levels, or whether different maternal loci are involved via a separate mechanism, we mapped specific maternal loci associated with neonatal cytokine/chemokines levels. We interestingly identified, via GWAS, three independent low-frequency significant maternal loci (MAF range 1–3%; *P* < 3.1 × 10^−8^) affecting three neonatal immune mediators. Neonatal levels of CCL24 were associated with the SNP rs72751339 in *MCTP2* on chromosome 15q26.2. The levels of IL-16 were associated with rs17159338 in the lincRNA *LINC01950* near the *EFNA5* gene (Ephrin-A5) on chromosome 5q21.3. Finally, CXCL5 (or ENA-78) was associated with rs34642455 in the cytochrome P450 3A4 gene (*CYP3A4*) on chromosome 7q22.1 (Table 2 and Additional file 3: Figure S2M–O). CCL24 showed distinct fetal- and maternal-associated loci whereas IL-16 and CXCL5 did not show significant fetal loci. These maternal SNPs were not associated with any maternal immune mediator (*P* < 0.05), except rs17159338 with IL-4 at *P* = 0.02.

### High fetal SNP-based contribution to maternal CXCL10 and IL-7

We also estimated whether the fetal genome affects maternal mid-gestational circulating cytokine/chemokine levels. About 99% of the maternal phenotypic variance of CXCL10 (SE = 37%, *P* = 5.8 × 10^−4^) and 90% of IL-7 (SE = 38%, *P* = 0.02) were explained by fetal heritability, compared to 79% and 84% maternal heritability estimates, respectively (Table 1). Once again, the high standard errors did not allow us to distinguish the exact maternal and fetal contributions or their independence. Thus, we estimated the proportion of the maternal circulating cytokine/chemokine levels regulated by fetal-specific alleles and by maternal-specific alleles. Maternal IL-7 and CXCL10 levels were significantly regulated by both maternal-specific alleles and fetal-specific alleles (Table 3).

### One fetal locus contributes to maternal soluble IL-2 receptor levels

We performed genome-wide association analysis using the fetal genome for each maternal mediator level. Supporting our heritability evidence of an active role of fetal genetics on the maternal immune system, the fetal locus rs12327057 (MAF = 18%) on chromosome 18p11 in RP11-78F17.1, a long noncoding RNA, near *ADCYAP1* (Fig. 2c and Table 2), was associated with soluble maternal interleukin-2 receptor levels (*β* = − 0.42, SE = 0.07, *P* = 1.4 × 10^−8^). The adenylate cyclase activating polypeptide 1 gene encodes secreted processed peptides involved in transcriptional activation of target genes. We did not measure sIL2R**-**α in neonates so we are not able to determine whether variation near the fetal *ADCYAP1* gene might affect the neonatal soluble immune receptor levels. However, maternal sIL2R**-**α levels were associated with variation at the *IL2RA* maternal locus, but not at the fetal *IL2RA* locus, supporting the interpretation that both maternal and fetal genetics might contribute to immune mediator variability, and specifically that fetal genetics influences maternal cytokines not via direct transfer of fetal-derived cytokines during pregnancy but via different pathways. This SNP was not associated with any neonatal immune mediator (*P* < 0.05), except with CCL24 at a nominal level (*P* = 5.0 × 10^−3^).

### Independent contribution of maternal- and fetal-associated loci to immune mediator variance

Most of the 17 genome-wide significant loci that were associated with cytokine/chemokine levels showed strong association when analysing either the contribution from fetal or maternal genetics but had reduced associations when considering paired mother/offspring genetics. We tested whether at these specific loci, fetal genetics contributes independently from maternal genetics to maternal immune mediators and whether fetal genetics contributes independently from maternal genetics to neonatal immune mediators. In nine out of 11 fetal genome-wide significant associated loci with maternal and neonatal immune mediators reported in Table 2, nominal maternal genetic association was observed; and in four out of six associated maternal loci with both maternal and fetal immune mediators, nominally significant fetal effects were seen (Table 2). When conditioning each associated immune mediator with both maternal and fetal genotypes, we observed no residual association in the mother/offspring with less significant initial evidence for association (Table 2 and Additional file 2: Table S9). Additionally, most of the maternal top SNPs (*P* < =5 × 10^−4^) do not overlap with the fetal top SNPs (*P* < =5 × 10^−4^) for the same immune mediators (Additional file 3: Figure S3).

### Interleukin-8 and CCL2 levels are associated with ASD outcome

**Fig. 3:**
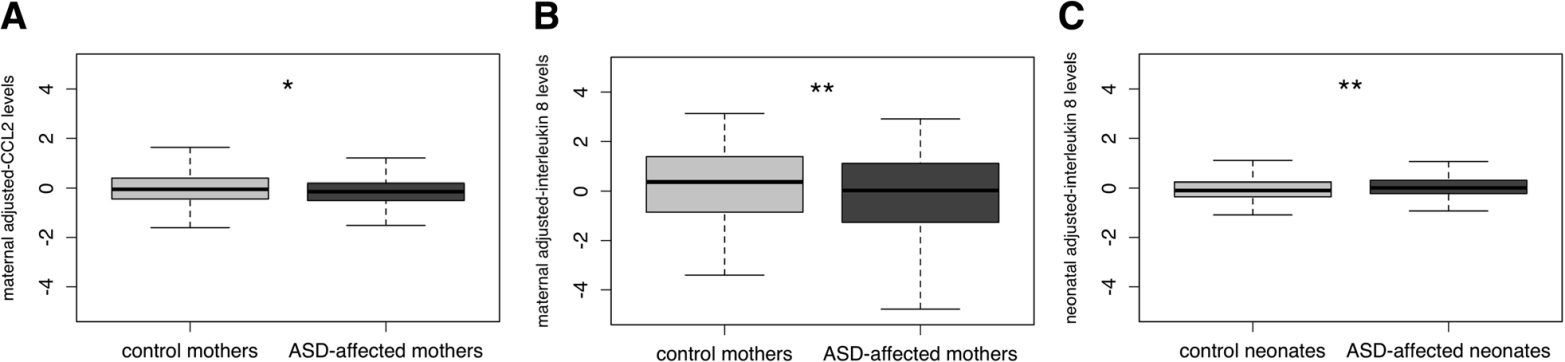
Association of maternal CCL2 (**a**) and maternal (**b**) and neonatal IL-8 (**c**) with ASD outcome. The level of each chemokine has been adjusted for the corresponding set of covariates. The levels of residuals for controls/mothers of controls (light grey) and ASD cases/mothers of ASD cases (dark grey) are shown

Consistent with previous findings using a quartile analysis approach in the full EMA sample [34, 47, Heuer LS, Jones KL, Yoshida CK, Hansen R, Yolken R, Zerbo O, Ashwood P, de WJ CLAV. An examination of neonatal cytokines and chemokines as predictors of autism risk: the early markers for autism study [In preparation]. We observed two borderline negative associations between maternal mid-gestational levels of the chemokines IL-8 and CCL2 (MCP1) with ASD (Wilcoxon test, *P* = 9.3 × 10^−3^ and *P* = 0.02, respectively) and a positive association between neonatal IL-8 and ASD (*P* = 8.6 × 10^−3^) (Table 4 and Fig. 3), after adjustment for the effects of covariates (Additional file 3: Figure S1). No additional significant association was detected.

**Table 4:** ASD outcome association with CCL2 and IL-8 in mothers and infants after adjustment for sociodemographic covariates and for maternal and fetal genetic ancestry

SIM	Dataset	*N*	Beta	SE	OR	[95% CI]	*P* value
CCL2	Mothers	707	− 0.135	0.058	0.874	[0.78–0.98]	0.019
IL-8	Mothers	707	− 0.243	0.112	0.784	[0.63–0.98]	0.030
IL-8	Infants	649	0.082	0.041	1.090	[1.00–1.18]	0.046

### Interleukin-8 levels interact with maternal genotype to show association with ASD

**Fig. 4:**
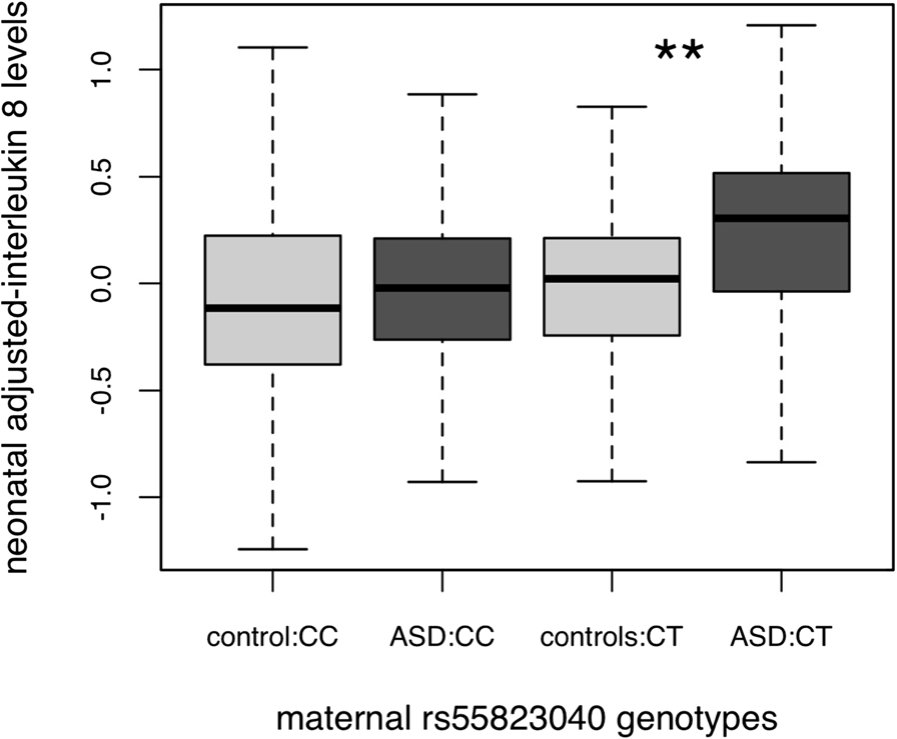
Neonatal residual levels of IL-8 showed interaction between ASD outcome and maternal SNP rs55823040. Controls are shown in light grey, and ASD cases in dark grey. Only CC and CT maternal genotype categories are shown. Few TT mothers are in EMA sample

We hypothesized that the specific genetic factors that contribute to CCL2 and IL-8 might drive the association observed with ASD outcome (Table 4). Since no genome-wide significant associated loci emerged for CCL2 and IL-8 (Table 2), we selected the most suggestively associated maternal and fetal SNPs: three maternal SNPs (maternal CCL2: rs1869714, *β* = 0.20, SE = 0.04, *P* = 4.3 × 10^−6^; maternal IL-8: rs60587996, *β* = − 0.77, SE = 0.16, *P* = 3.1 × 10^−6^, neonatal IL-8: rs55823040, *β* = 0.24, SE = 0.05, *P* = 3.6 × 10^−7^) and three fetal SNPs (maternal CCL2: rs17504601, *β* = 0.28, SE = 0.06, *P* = 5.0 × 10^−7^; maternal IL-8: rs1252145, *β* = − 0.66, SE = 0.13, *P* = 5.3 × 10^−7^, neonatal IL-8: rs80166972, *β* = 0.47, SE = 0.09, *P* = 4.3 × 10^−7^). These SNPs were not themselves associated in logistic models with ASD outcome (*P* > 0.1), but we observed a significant interaction (*β* = − 0.22 SE = 0.11, *P* = 0.04) between ASD outcome and maternal SNP rs55823040 (MAF = 0.09), which was associated (*P* = 3.6 × 10^−7^) with neonatal IL-8 levels. Our analysis showed that neonatal IL-8 levels were significantly increased only in ASD offspring of mothers with the CT genotype for rs55823040 (Fig. 4) (few TT homozygotes were observed). This SNP had only interaction effects and not main effects with ASD outcome.

## Discussion

**Fig. 5:**
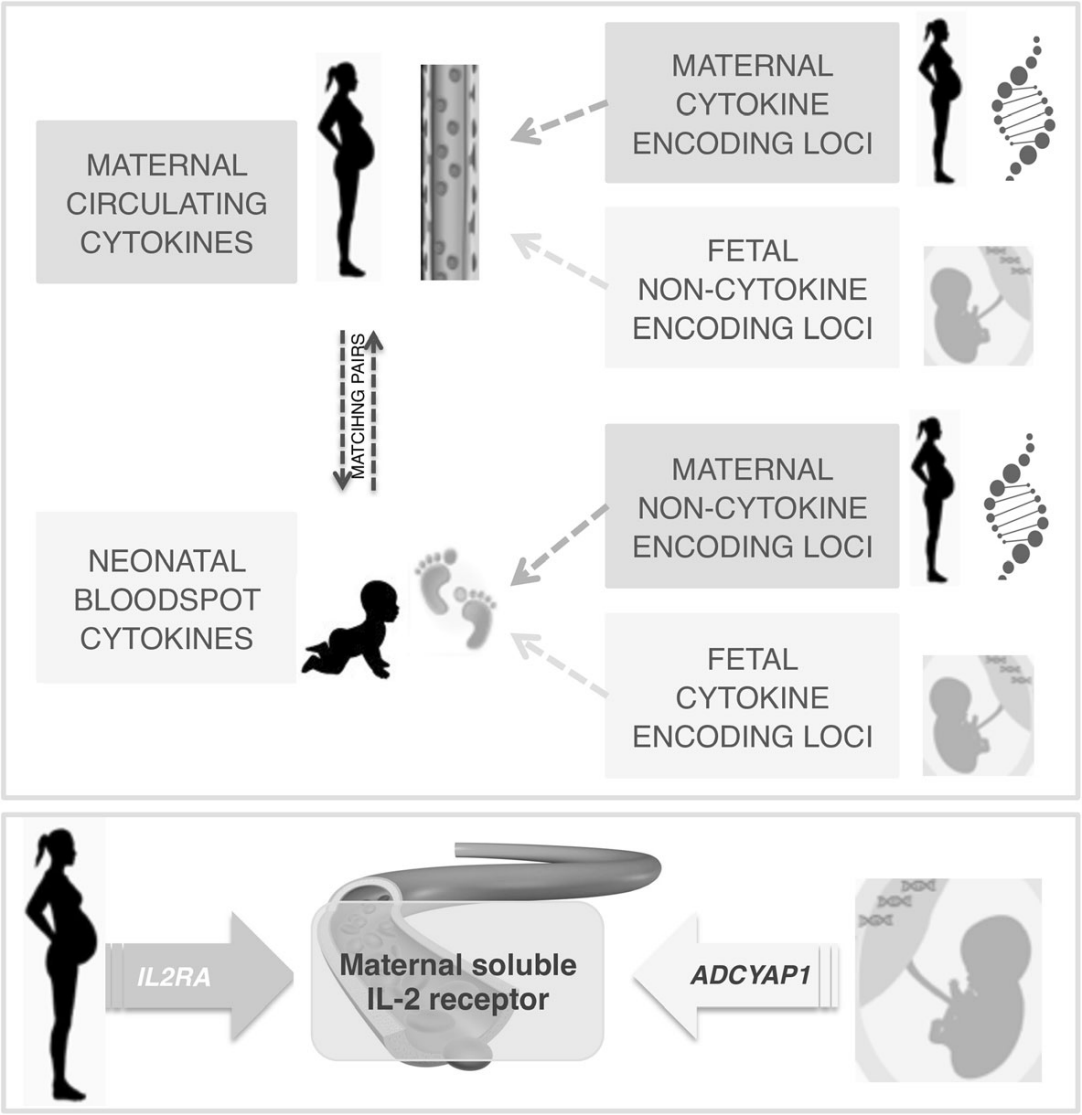
Outline. In the first panel, the diagram of the multi-approach analysis and mechanisms identified are shown. In the second panel, the serum levels of maternal sIL2-Ra are regulated by maternal genetics through cytokine receptor-encoding gene (*IL2RA*) and by fetal genetics through a non-cytokine-encoding gene (*ADCYAP1*) not previously involved in immune system status

We report here the first genome-wide multi-approach study to provide insight into potential patterns of maternal-fetal genetic control of immune mediator status in the prenatal and neonatal periods (Fig. 5). We discuss specific significant findings, some of which may be unique to these life stages. In addition, we detail our observations supporting the interpretation that cross-genetic associations represent independent maternal and fetal influence on one another and that this influence occurs via a distinct mechanism rather than direct exchange of soluble factors.

We showed that the circulating levels of CXCL10 (IP-10) and IL-7 during mid-gestation were strongly regulated by the maternal genome. These results were consistent with two studies analysing the SNP-based heritability and twin-based heritability of immune molecules [48, 49]. Many of the immune mediators without significant estimated genetic contribution showed significant GWAS associations, suggesting genetic influence beyond what we could detect as heritability. We identified three maternal loci that were potentially involved in mid-gestational immune system status. A SNP (rs12778662) was associated with soluble levels of IL-2 receptor alpha and maps near the gene encoding *IL2RA* (Fig. 5). This association has previously been observed in healthy adults [8]. In addition, the chemokine CCL11, a strong activator of eosinophils, was associated with a novel SNP located between *THRB (*encoding the thyroid hormone receptor *β*) and *RARB (*encoding retinoic acid receptor *β*), which is effective at controlling inflammatory and inducing tolerogenic immune responses. Finally, maternal levels of the inflammatory cytokine IL1-α were associated with a novel SNP near *SMAD1,* which encodes one of the SMAD proteins involved in the TGF-*β* signalling pathway. These latter two associations were not observed (*P* > 0.05) in large populations of healthy adults studied [8].

We also assessed neonatal cytokine/chemokine levels and their potential genetic control in early life. The reduced variance observed in the distributions of neonatal cytokines suggests that stressful events during birth did not unduly impact neonatal immune mediator levels, allowing for the detection of genetic influence. Moreover, there were low correlations between neonatal and maternal cytokine/chemokine levels. Although the samples are taken months apart, should they reflect baseline genetically-determined levels, we might expect correlation if neonatal immune mediators were directly transferred from the maternal bloodstream. Thus, our observations suggest that maternal cytokines and chemokines are not directly populating the neonatal blood at birth. However, since the neonatal levels reflect both serum and lysed cells, and the maternal levels were measured only in serum, the levels measured may not be directly comparable. Note that the difference in variation observed in maternal and neonatal immune mediator distributions may be biological variation (e.g. influence of long-term environmental exposure in mothers) or it may be technical noise (e.g. serum vs. bloodspot or different assay properties); however, either of these explanations are non-genetic and thus will have the same impact on our genetic analyses.

Genome-wide association analysis identified ten fetal loci associated with 19 circulating neonatal immune mediators at birth (Table 2). We defined a set of six neonatal chemokines, CCL1, CCL3, CCL17, CCL19, CCL22, and CCL25, controlled by shared common fetal genetic factors. Interestingly, a single large-impact SNP (variance explained about 13%) rs75885714 was associated with CXCL9, which drives the association with ten other highly correlated (*ρ* > 0.90) neonatal cytokines/chemokines including the heritable CCL17 and CCL19. The marker rs75885714 maps in *PLCL2,* which encodes a phospholipase involved in the regulation of calcium-protein kinase C signalling pathway. In studies of *Plcl2*-deficient mice, it has been shown that *Plcl2* acts as a negative regulator of B cell receptor signalling and humoral immune responses [50], and in humans is involved in metabolism [51] and implicated in autoimmune diseases such as psoriasis [52], rheumatoid arthritis [53], and systemic sclerosis [54]. However, this is the first study to show *PLCL2* associated with quantitative levels of immune molecules, which could reflect a developmental observation.

Seven other fetal loci associated with neonatal cytokine and chemokine levels were located in the gene encoding the receptor of that cytokine/chemokine. Among these seven loci, our study found evidence for association with a cluster of atypical chemokine receptors (ACKRs) and confirms evidence shown in a recently published study [8]. The study analysing an adult human population from Finland showed association between *ACKR1* and *ACKR2* and other immune molecules but did not replicate our neonatal finding in *ACKR4*. Our neonatal EMA sample replicates at a nominally significant level the association of *ACKR1* (CXCL11; *P* = 9.0 × 10^–3)^ in adults, but we did not replicate all previously-implicated SNPs [8], likely due to differences between the two studies, such as the age of the recruited participants and/or the genetic make-up of the Finnish population.

Finally, two other fetal SNPs were identified in loci not attributable so far to any known immune function. In particular, one SNP that was associated with neonatal IL-4 levels, maps near to *TDRP*, a gene that encodes a testis development related protein that is expressed in the thyroid gland and placenta, and has not previously been associated with immune phenotypes in adults. Finally, CXCL12 was associated with a SNP in a lincRNA between *LSAMP* (limbic system-associated membrane protein) and *IGSF11* (immunoglobulin superfamily member 11), both involved in cell-cell adhesion and may be important in immune synapse formation. Although this SNP has not been associated at a genome-wide significant level with cytokines/chemokines previously, it did show modest signal with several molecules (*P*_*min*_ = 8 × 10^−4^) [8].

Since the circulating levels of mid-gestational cytokines and chemokines were not correlated with neonatal levels, we asked whether maternal genetics might directly regulate neonatal cytokines/chemokines at birth. We found significant maternal genetic contribution for six neonatal immune mediators, suggesting a combination of independent maternal and neonatal genetic control for most of those. IL-4 showed only maternal evidence for heritability. In pregnancy, IL-4 is produced [55] by immune cells of the placenta, by the maternal decidua, amniochorionic membranes, cytotrophoblasts, and both maternal and fetal endothelial cells. IL-4 is an archetypal cytokine involved in TH2-mediated adaptive immunity and during pregnancy is thought to help reduce the risk of miscarriage. Our result suggests that neonatal IL-4 levels might be actively regulated by maternal genetics. However, what those genetic mechanisms are, we were unable to determine. We found only one maternal SNP (rs7546782) that was significantly associated with neonatal IL-4 at borderline levels (*P* = 7.7 × 10^−8^) and maps to a region in chromosome 1 that is rich in regulatory elements (see the full set of summary statistics-level results available at DOI: 10.5281/zenodo.1321338).

Three maternal GW-associated loci regulate neonatal mediator levels. The neonatal chemokine CCL24 is associated with a maternal SNP in *MCTP2*, which encodes a transmembrane protein that binds to Ca^2+^. MCTP2 is involved in intercellular signal transduction and synapse function and potentially metabolic processes as it is associated with body mass index [56, 57]. Variation near maternal *EFNA5*, a member of the ephrin family, is associated with the neonatal cytokine IL-16. Ephrin ligands and receptors are observed in lymphocytes, monocytes, and dendritic cells, and their expression levels change during inflammation. Finally, the neonatal chemokine CXCL5 is associated with a maternal locus in the cytochrome P450 3A4 gene (*CYP3A4*), one of the most important effectors of oxidative metabolism in humans. None of these loci have previously been associated with adult immune phenotypes [8].

Previous evidence showed that fetal genetics could also regulate several maternal phenotypes during pregnancy [10, 11]. In our sample, maternal IL-7 and CXCL10 were under the control of independent contributions from maternal-specific and fetal-specific non-overlapping alleles. We further identified one fetal locus associated with maternal sIL2R-α, *ADCYAP1*, or *PACAP*, which encodes a signalling neuropeptide widely expressed in the central and peripheral nervous systems. PACAP is also expressed in different parts of the placenta and the umbilical cord where it affects cell survival, angiogenesis, and proliferation of trophoblast cells [58]. This locus was not associated with any fetal cytokine/chemokine, nor has it previously been associated with adult immune phenotypes [8].

Across our results, the cross-genetic associated loci map to genes that were not already associated with the analysed cytokines/chemokines and they showed independent association with only maternal or fetal genetics, but the same locus never showed evidence for both maternal and fetal effects. As summarized in Fig. 5, our genetic data suggest that the cross-genetic influence between mother and fetus is not simply a redistribution of soluble immune mediators, in which case we would have expected to see the same loci contributing to the same mediator in both genetic datasets, and that these would predominantly encode genes directly relevant to each cytokine/chemokine or its receptor. Our alternative hypothesis (Fig. 5) is that most of the maternal-fetal impact occurs via distinct regulatory or indirect placental mechanisms such as signalling at the placental interface and through a different set of genes than currently implicated in inflammation. Moreover, these mechanisms could potentially be responsible for the pregnancy-specific regulation of the cytokines/chemokines produced by maternal and/or fetal placental tissues within the tissues of pregnancy [59] and possibly in the peripheral blood.

Finally, we observed that maternal levels of the chemokines IL-8 and CCL2 were significantly lower in mothers of ASD-affected children compared to mothers of control children. We also identified a positive association with neonatal IL-8 levels that is consistent with our findings in a larger EMA sample. Other studies implicating IL-8 include measuring IL-8 in the plasma of young (2–5 years of age) ASD children [19] and one showing increased expression of the IL-8 gene in brains of ASD children [60]. Interestingly*,* we demonstrated that the neonatal levels of IL-8 were significantly associated with ASD outcome only in children of mothers heterozygous for SNP rs55823040, which was suggestively associated with overall neonatal IL-8 levels. This SNP maps near the pseudogene *CACYBPP2*, between the nucleoporin *NUP35* and the zinc finger protein ZNF804A, a candidate for ASD, schizophrenia [61], and other neuropsychiatric disorders [62]. This SNP did not show main effects with ASD outcome but only the observed interaction with IL-8 levels. It will be important to replicate this finding in additional studies.

This study has several limitations, including sample size and lack of information about infection or illness at the time of sampling. The population sampled also includes heterogeneous genetic ancestries. While this introduces methodological challenges and does not allow for heritability estimates precise for a homogenous population, we have used a number of approaches to ensure that our study design did not introduce false positives into our results. Overall, we believe studying a representative US population (Southern California) is a significant strength of this study, as our results are more likely to be applicable across genetic populations.

## Conclusions

Taking advantage of a unique set of mother-infant matching pairs, this study shows patterns of genetic variation regulating cytokine/chemokine status during pregnancy and at birth. We identified a total of 17 specific maternal and fetal loci contributing to one or more cytokine and chemokine status at the two different time points. Several of these are novel and may be shown to be specific to pregnancy and newborn periods upon further study. Our cross-genetic analysis identified distinct maternal loci in novel regions independently associated with cytokine/chemokine levels in our neonatal dataset and that fetal loci in novel regions were independently associated with maternal cytokine/chemokine levels. We speculate that this pattern of cross-genetic influences at non-cytokine, non-chemokine, and non-receptor encoding loci and exclusively at loci not previously implicated for adult immune molecule levels implies a major influence of variation in placental biology on cytokine and chemokine levels. Interestingly, the observation of an association between increased IL-8 and ASD status only in the presence of a specific maternal SNP genotype suggests that future studies should consider the cross-genetic interaction involved in early risk for neurodevelopmental disorders.

## Additional files

Additional file 1 Supplemental methods. Neonatal filter extraction and validity of the multiplex assay for maternal and neonatal immune mediators. (PDF 42 kb)

Additional file 2 **Table S1.** Statistics for 22 maternal immune mediator levels and 42 neonatal immune mediator levels after adjustment for individual-specific confounding factors. **Table S2.** Spearman’s correlation coefficient (φ) across ten maternal principal coordinates and ten fetal principal coordinates. **Table S3.** Statistics for 22 maternal immune mediator levels and 42 neonatal immune mediator levels after adjustment for individual-specific confounding factors. **Table S4.** Spearman’s correlation coefficient (φ) across cytokines and chemokines within mothers. **Table S5.** Spearman’s correlation coefficient (φ) across cytokines and chemokines within infants. Table S6. Spearman’s correlation coefficient (φ) across 15 overlapping immune molecules measured in maternal and neonatal datasets. **Table S7.** Significant genetic correlation across heritable covariate-adjusted neonatal immune cytokine/chemokine levels and corresponding linear correlation. **Table S8.** Comparison of maternal and fetal genome-wide significant associations (Table 2) and meta-analysis across populations. **Table S9.** Combined effects of maternal-fetal SNPs in linear regression models. (XLSX 836 kb)

Additional file 3 **Figure S1.** Significance levels of each confounding factor across the entire set of maternal and neonatal immune mediators. **Figure S2.** Linkage disequilibrium regional genomic plots. **Figure S3.** Maternal and fetal SNPs (*P* < = 5 × 10^–4^) from maternal sIL2R-a and neonatal CCL24 summary statistics. (PDF 2347 kb)

## Abbreviations

ASD: Autism spectrum disorder
chr: Chromosome
CI: Confidence interval
EMA: Early Markers for Autism
gen: Genetics
GWAS: Genome-wide association study
h^2^_g_: SNP-based heritability
I: Infants
LD: Linkage disequilibrium
LOD: Limit of detection
M: Mothers
MAF: Minor allele frequency
MDS: Multidimensional scaling
MIA: Maternal immune activation
OR: Odds ratio
SE: Standard error
SIM: Soluble immune mediator
