# Supplementary material for: Cross-genetic determination of maternal and neonatal immune mediators during pregnancy
Source: Genome Med. 2018 Aug 22;10:67. doi: 10.1186/s13073-018-0576-8 (PMC6106874; doi:10.1186/s13073-018-0576-8)
Supplement: Supplementary file 1 — Supplemental methods. Neonatal filter extraction and validity of the multiplex assay for maternal and neonatal immune mediators. (PDF 42 kb) [file 13073_2018_576_MOESM1_ESM.pdf]

Supplemental Methods

Neonatal filter extraction

The neonatal dried blood spots were collected on filter paper by 72 hours and stored at 20°C for an extended but similar period of time (10 years approximately) prior to the analysis so the degree of degradation was comparable across all sample types [Heuer et al., in submission]. They were maintained by the Genetic Disease Screening Program, California Department of Public Health, then they were received on dry ice in 96-well plates as 3x3mm punches per subject in single wells of the plate, and stored at -80°C until elution. The day prior to assaying for cytokines/chemokines, each sample received 200µl of elution buffer (.5% BSA in 50ml PBS with 1 tablet of Roche Complete Protease Inhibitor Cocktail; Roche Applied Science, Indianapolis, IN) and was placed on a plate shaker overnight at 4°C. The following morning eluates were isolated from the filter paper spots and a small 4µl aliquot used for BCA assay (Thermo Scientific, Rockford, IL) determination of total protein to normalize cytokine/chemokine levels against blood sample quantity variation. Immediately following overnight elution, neonatal levels of peripheral blood immune markers were determined using a commercially available, slightly modified, Luminex multiplex magnetic bead assay as described in Methods.

Validity of the multiplex extraction for maternal and neonatal immune mediators

Rigorous assay validation including linearity of dilution (around 98% - 99%), recovery, sensitivity and specificity were reported in the panel sheets (see neonatal assay details at http://www.bio-rad.com/webroot/web/pdf/lsr/literature/Bulletin_6499.pdf and maternal assay details at http://www.filgen.jp/Product/Bioscience19-Bioplex/HCYTOMAG-60K.MPX.pdf). Also, reference samples were used on each plate to ensure assay consistency.
