# Supplementary material for: Cross-genetic determination of maternal and neonatal immune mediators during pregnancy
Source: Genome Med. 2018 Aug 22;10:67. doi: 10.1186/s13073-018-0576-8 (PMC6106874; doi:10.1186/s13073-018-0576-8)
Supplement: Supplementary file 3 — Figure S1. Significance levels of each confounding factor across the entire set of maternal and neonatal immune mediators. Figure S2. Linkage disequilibrium regional genomic plots. Figure S3. Maternal and fetal SNPs (P < = 5 × 10^–4^) from maternal sIL2R-a and neonatal CCL24 summary statistics. (PDF 2347 kb) [file 13073_2018_576_MOESM3_ESM.pdf]

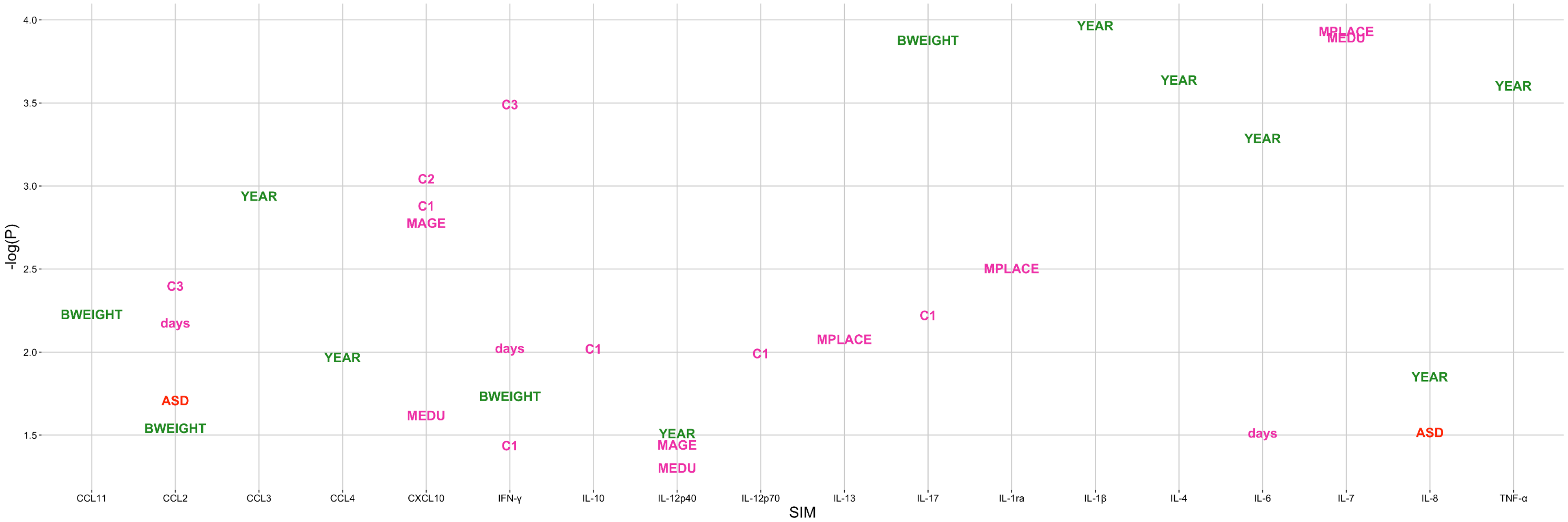

-log(P)
4.0
3.5
3.0
2.5
2.0
1.5
BWEIGHT
C3
days
ASD
BWEIGHT
YEAR
YEAR
C2
C1
MAGE
MEDU
C3
days
BWEIGHT
C1
C1
YEAR
MAGE
MEDU
C1
MPLACE
BWEIGHT
C1
MPLACE
YEAR
YEAR
YEAR
days
MPLACE
MEDU
YEAR
ASD
YEAR
CCL11
CCL2
CCL3
CCL4
CXCL10
IFN-γ
IL-10
IL-12p40
IL-12p70
IL-13
IL-17
IL-1ra
IL-1β
IL-4
IL-6
IL-7
IL-8
TNF-α
SIM

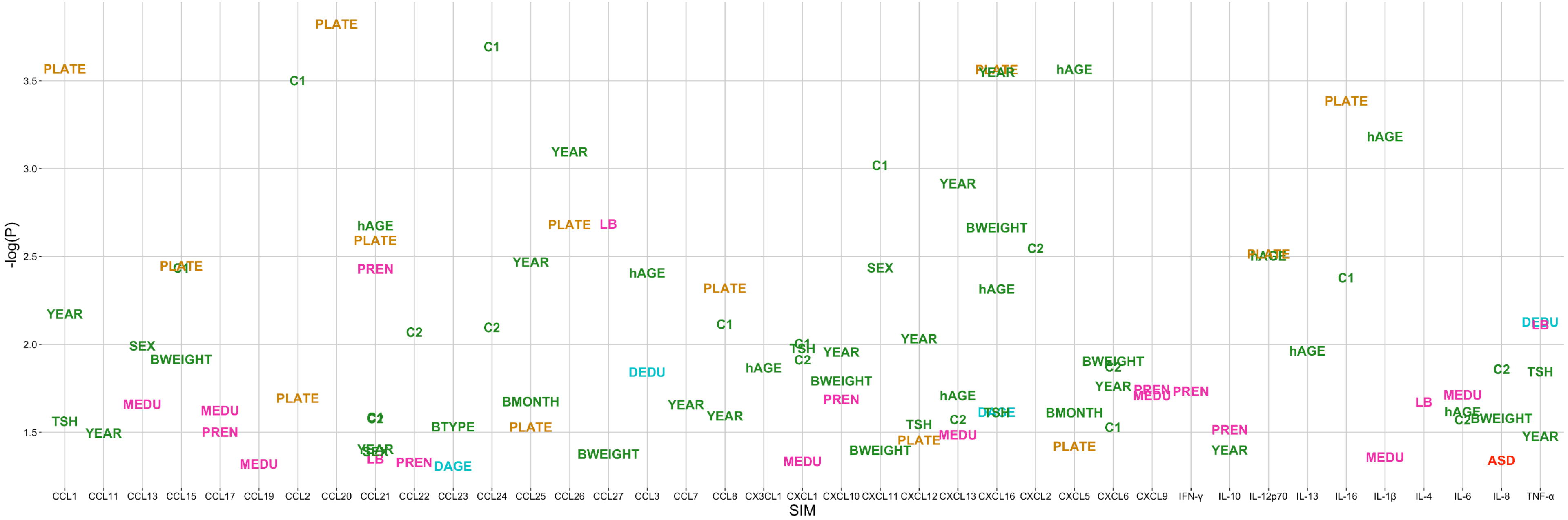

-log(P)
3.5
3.0
2.5
2.0
1.5
PLATE
YEAR
TSH
YEAR
SEX
MEDU
PLATE
BWEIGHT
MEDU
PREN
MEDU
C1
PLATE
PLATE
hAGE
PLATE
PREN
C1
YEAR
LB
PREN
C2
BTYPE
DAGE
C1
C2
YEAR
BMONTH
PLATE
YEAR
PLATE
LB
BWEIGHT
hAGE
DEDU
YEAR
PLATE
C1
YEAR
hAGE
C1
TSH
C2
MEDU
YEAR
BWEIGHT
PREN
C1
SEX
BWEIGHT
YEAR
TSH
PLATE
YEAR
hAGE
C2
MEDU
PLATE
YEAR
BWEIGHT
hAGE
TSH
C2
hAGE
BMONTH
PLATE
BWEIGHT
C2
YEAR
C1
PREN
MEDU
PREN
PREN
YEAR
PLATE
hAGE
hAGE
PLATE
C1
hAGE
MEDU
LB
MEDU
hAGE
C2
BWEIGHT
C2
ASD
DEDU
LB
TSH
YEAR
CCL1
CCL11
CCL13
CCL15
CCL17
CCL19
CCL2
CCL20
CCL21
CCL22
CCL23
CCL24
CCL25
CCL26
CCL27
CCL3
CCL7
CCL8
CX3CL1
CXCL1
CXCL10
CXCL11
CXCL12
CXCL13
CXCL16
CXCL2
CXCL5
CXCL6
CXCL9
IFN-γ
IL-10
IL-12p70
IL-13
IL-16
IL-1β
IL-4
IL-6
IL-8
TNF-α
SIM

Fig. S1 Significance levels of each confounding factor across the entire set of maternal and neonatal immune mediators. (**A**) The plot shows the significance level of covariates that reached $P<0.1$ in each of 22 maternal cytokine/chemokine models (**B**). The plot shows the significance level of covariates that reached $P<0.1$ in each of 42 neonatal cytokine/chemokine models. X-axis shows each SIM, Y-axis show –log(P-value). The covariates that come from neonates are shown in green, from mothers in pink, from fathers are in lightblue, ASD in red is significant for maternal CCL2 (P=0.019) and maternal/neonatal IL-8 (P=0.030 and P=0.045, respectively), PLATE, to control for potential technical differences related to the experiment, is in orange. Abbreviations: ASD: offspring affection status; BWEIGHT: neonatal birth weight; BMONTH: neonatal birth month; YEAR: neonatal birth year; BTYPE: neonatal birth type; SEX: neonatal gender; hAGE: hour after the birth at bloodspot time; TSH: neonatal TSH; PREN: maternal number of prenatal visits; LB: maternal live births; days: maternal gestational age at blood draw; MAGE: maternal age; MEDU: maternal education; DAGE: father age; DEDU: father education; PLATE: plate number; C1: principal component 1; C2: principal component 2; C3: principal component 3; Seven additional principal components used in each model are not shown.

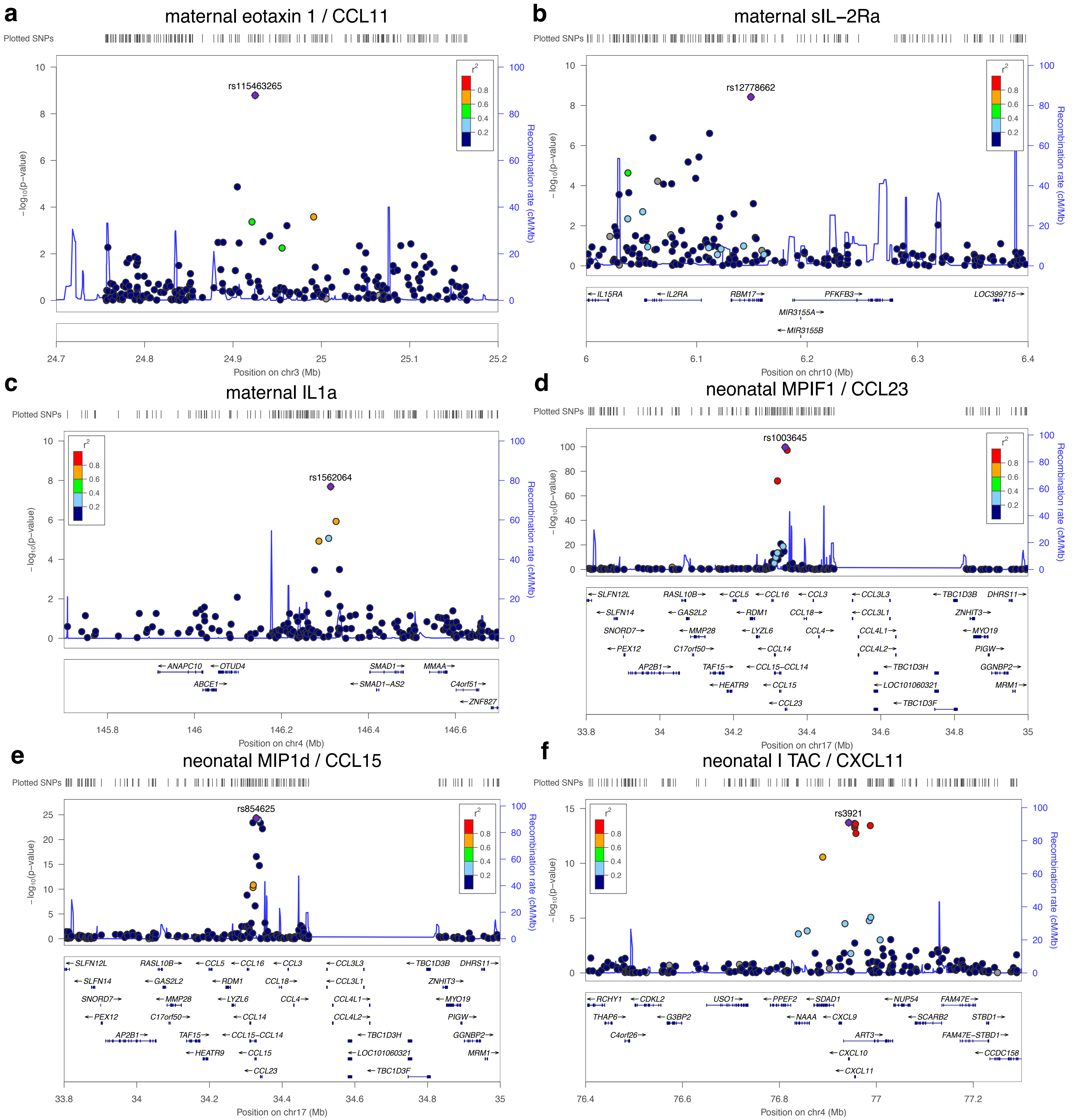

a
maternal eotaxin 1 / CCL11
Plotted SNPs
rs115463265
Position on chr3 (Mb)
b
maternal sIL−2Ra
rs12778662
IL15RA
IL2RA
RBM17
PFKFB3
LOC399715
MIR3155A
MIR3155B
Position on chr10 (Mb)
c
maternal IL1a
rs1562064
ANAPC10
OTUD4
ABCE1
SMAD1
SMAD1−AS2
MMAA
C4orf51
ZNF827
Position on chr4 (Mb)
d
neonatal MPIF1 / CCL23
rs1003645
Position on chr17 (Mb)
e
neonatal MIP1d / CCL15
rs854625
Position on chr17 (Mb)
f
neonatal I TAC / CXCL11
rs3921
RCHY1
CDKL2
USO1
PPEF2
SDAD1
NUP54
FAM47E
THAP6
G3BP2
NAAA
CXCL9
SCARB2
STBD1
C4orf26
ART3
FAM47E−STBD1
CXCL10
CCDC158
CXCL11
Position on chr4 (Mb)
−log10(p−value)
Recombination rate (cM/Mb)

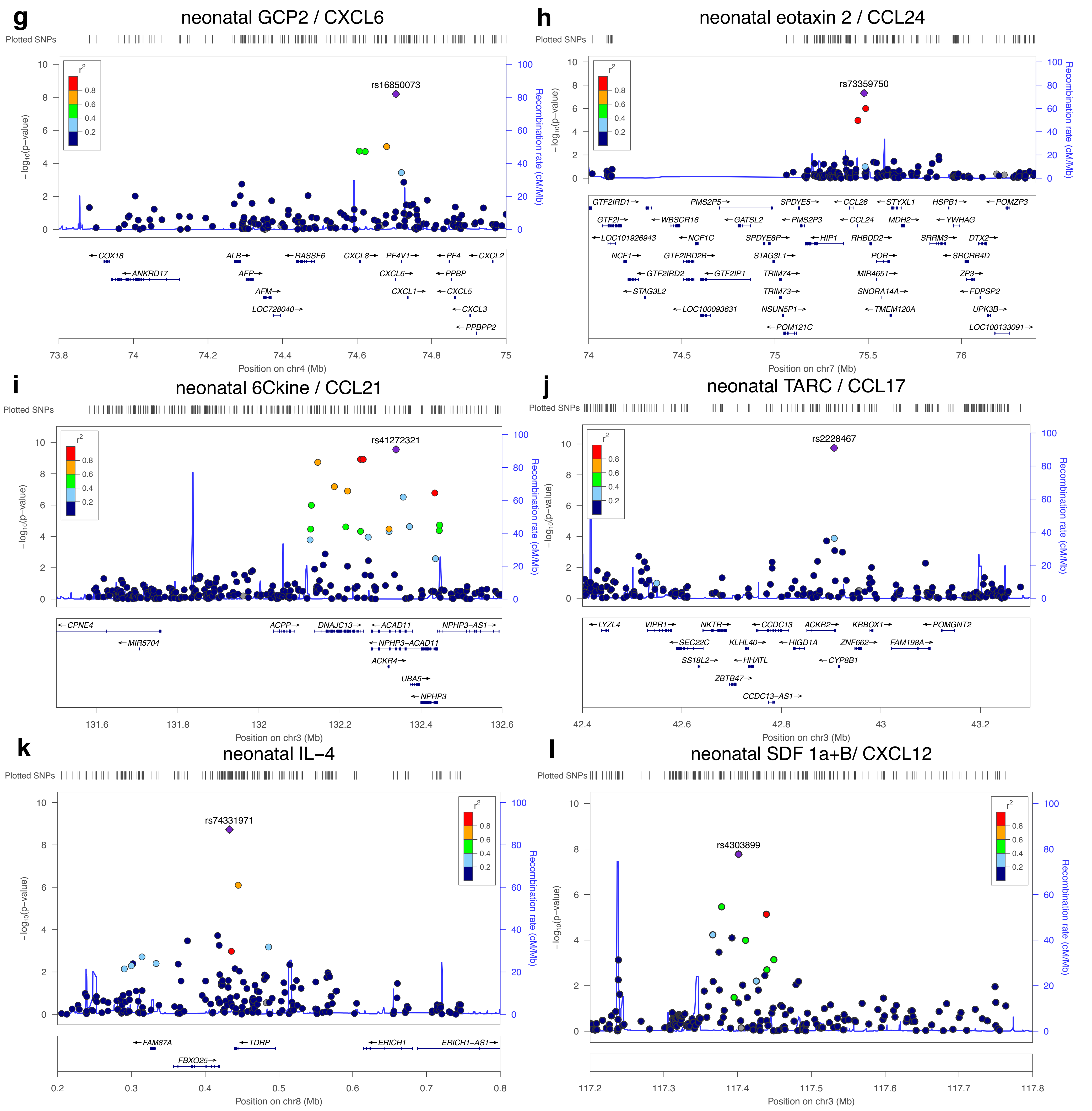
g
neonatal GCP2 / CXCL6
Plotted SNPs
rs16850073
r2
0.8
0.6
0.4
0.2
−log10(p−value)
Recombination rate (cM/Mb)
COX18
ANKRD17
ALB
AFP
AFM
LOC728040
RASSF6
CXCL8
PF4V1
CXCL6
CXCL1
PF4
PPBP
CXCL5
CXCL3
PPBPP2
CXCL2
Position on chr4 (Mb)
h
neonatal eotaxin 2 / CCL24
Plotted SNPs
rs73359750
GTF2IRD1
GTF2I
LOC101926943
NCF1
GTF2IRD2
STAG3L2
PMS2P5
WBSCR16
NCF1C
GTF2IRD2B
LOC100093631
GATSL2
GTF2IP1
SPDYE5
PMS2P3
SPDYE8P
STAG3L1
TRIM74
TRIM73
NSUN5P1
POM121C
HIP1
CCL26
CCL24
RHBDD2
POR
MIR4651
SNORA14A
TMEM120A
STYXL1
MDH2
HSPB1
YWHAG
SRRM3
SRCRB4D
ZP3
FDPSP2
UPK3B
LOC100133091
POMZP3
DTX2
Position on chr7 (Mb)
i
neonatal 6Ckine / CCL21
Plotted SNPs
rs41272321
CPNE4
MIR5704
ACPP
DNAJC13
ACAD11
NPHP3-ACAD11
ACKR4
UBA5
NPHP3
NPHP3-AS1
Position on chr3 (Mb)
j
neonatal TARC / CCL17
Plotted SNPs
rs2228467
LYZL4
VIPR1
SEC22C
SS18L2
NKTR
KLHL40
HHATL
ZBTB47
CCDC13
HIGD1A
CCDC13-AS1
ACKR2
CYP8B1
KRBOX1
ZNF662
POMGNT2
FAM198A
Position on chr3 (Mb)
k
neonatal IL−4
Plotted SNPs
rs74331971
FAM87A
FBXO25
TDRP
ERICH1
ERICH1-AS1
Position on chr8 (Mb)
l
neonatal SDF 1a+B/ CXCL12
Plotted SNPs
rs4303899
Position on chr3 (Mb)

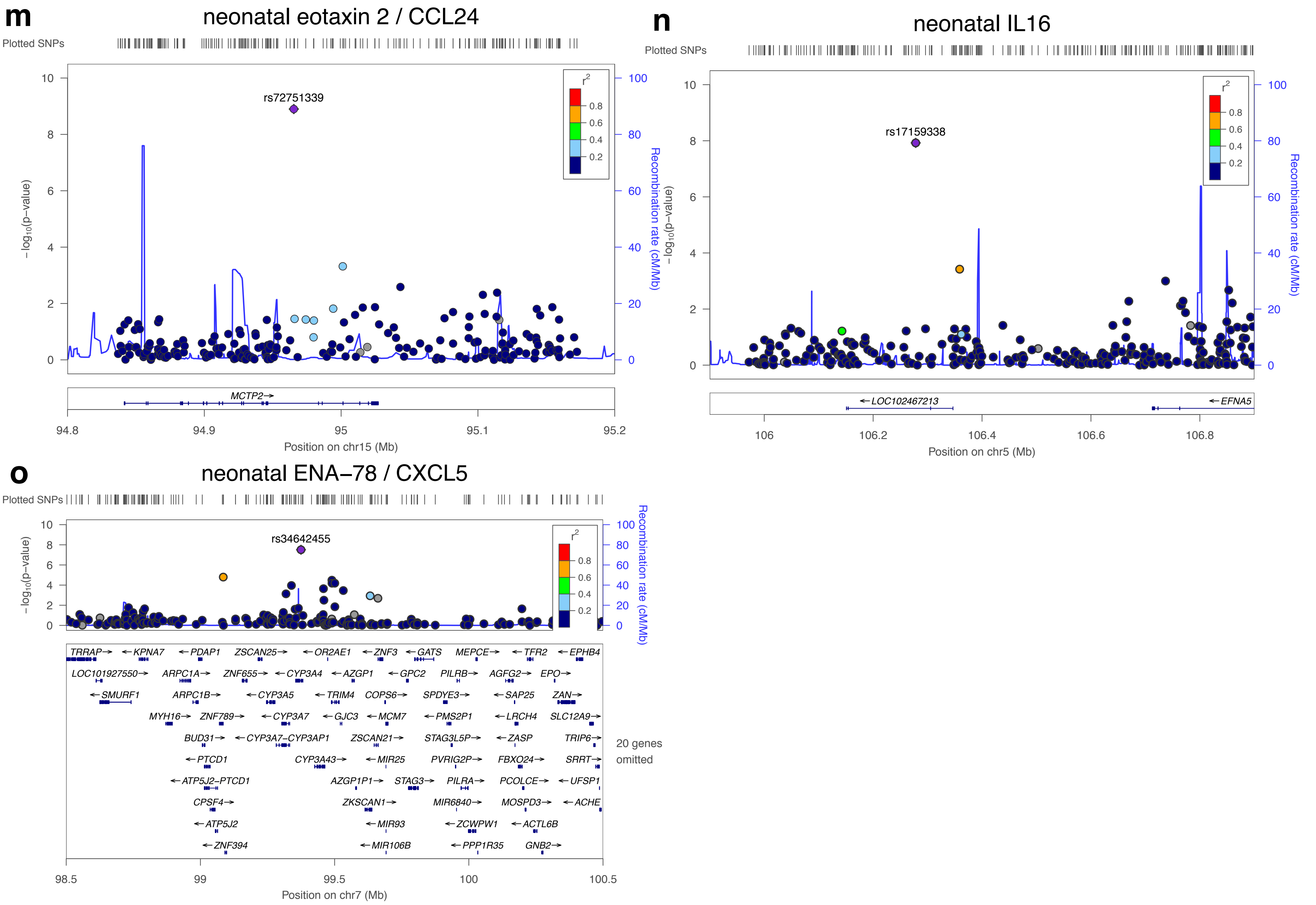
m
neonatal eotaxin 2 / CCL24
Plotted SNPs
rs72751339
r2
−log10(p−value)
Recombination rate (cM/Mb)
MCTP2→
Position on chr15 (Mb)
n
neonatal IL16
Plotted SNPs
rs17159338
r2
−log10(p−value)
Recombination rate (cM/Mb)
←LOC102467213
←EFNA5
Position on chr5 (Mb)
o
neonatal ENA−78 / CXCL5
Plotted SNPs
rs34642455
r2
−log10(p−value)
Recombination rate (cM/Mb)
TRRAP→ ←KPNA7 ←PDAP1 ZSCAN25→ ←OR2AE1 ←ZNF3 ←GATS MEPCE→ ←TFR2 ←EPHB4
LOC101927550→ ARPC1A→ ZNF655→ ←CYP3A4 ←AZGP1 ←GPC2 PILRB→ AGFG2→ EPO→
←SMURF1 ARPC1B→ ←CYP3A5 ←TRIM4 COPS6→ SPDYE3→ ←SAP25 ZAN→
MYH16→ ZNF789→ ←CYP3A7 ←GJC3 ←MCM7 ←PMS2P1 ←LRCH4 SLC12A9→
BUD31→ ←CYP3A7−CYP3AP1 ZSCAN21→ STAG3L5P→ ←ZASP TRIP6→
←PTCD1 CYP3A43→ ←MIR25 PVRIG2P→ FBXO24→ SRRT→
←ATP5J2−PTCD1 AZGP1P1→ STAG3→ PILRA→ PCOLCE→ ←UFSP1
CPSF4→ ZKSCAN1→ MIR6840→ MOSPD3→ ←ACHE
←ATP5J2 ←MIR93 ←ZCWPW1 ←ACTL6B
←ZNF394 ←MIR106B ←PPP1R35 GNB2→
20 genes omitted
Position on chr7 (Mb)

Fig. S2 Linkage disequilibrium regional genomic plots. Maternal genome-wide associated SNPs with maternal immune mediators (**A-C**); fetal genome-wide associated SNPs with neonatal immune mediators (**D-L**) and maternal genome-wide associated SNPs with neonatal immune mediators (**M-O**). The X-axis represents the genomic position; the Y-axis shows the negative logarithm of the observed association p-value for each tested SNP. Plotted with Locuzoom tool[42]

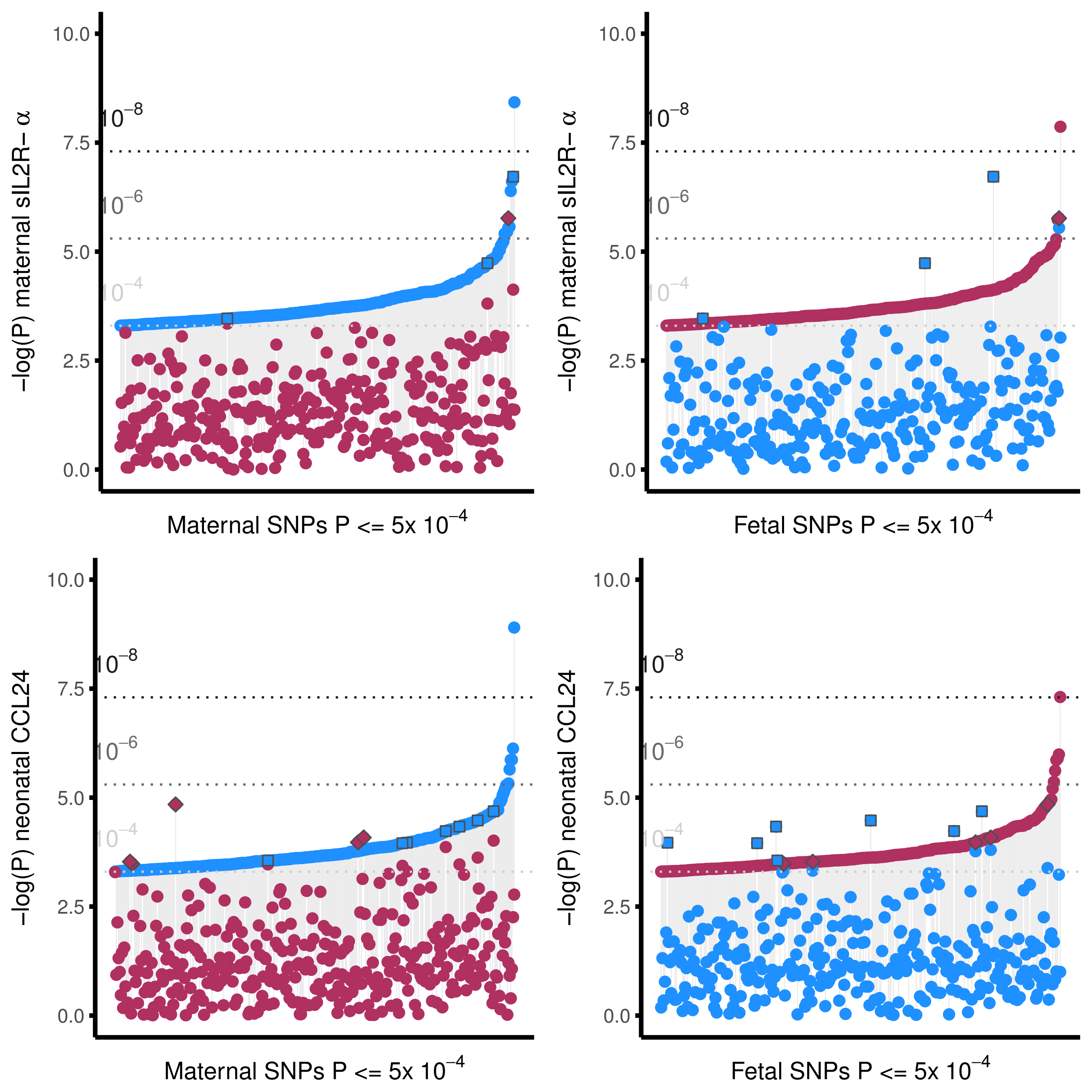

-log(P) maternal sIL2R- α
Maternal SNPs P <= 5x 10^{-4}
Fetal SNPs P <= 5x 10^{-4}
-log(P) neonatal CCL24
10^{-8}
10^{-6}
10^{-4}
10.0
7.5
5.0
2.5
0.0

Fig. S3 Maternal and fetal SNPs (*P* <= 5x10^{-4}) from maternal sIL2R-a and neonatal CCL24 summary statistics. (**A**) Maternal ranked SNPs associated with maternal sIL2Ra and the corresponding fetal SNP association; (**B**) fetal ranked SNPs associated with maternal sIL2-Ra and the corresponding maternal SNP association; (**C**) maternal ranked SNPs associated with neonatal CCL24 and the corresponding fetal SNP association and (**D**) fetal ranked SNPs associated with neonatal CCL24 and the corresponding maternal SNP association. The X-axis represents the ranked SNPs based on the observed $P <= 5x10^{-4}$ in one individual's genetics (maternal/fetal); the Y-axis shows the negative logarithm of the observed p-value for each tested SNP. The maternal SNPs and the fetal SNPs are shown in 'light blue' and 'purple', respectively. The SNPs that showed $P <= 5x10^{-4}$ in both individuals are represented with a purple diamond when fetal-driven and with a light blue square when maternal-driven. No SNPs showed significant independent association with both individuals.
